# A conditionally replication-defective cytomegalovirus vaccine elicits potent and diverse functional monoclonal antibodies in a phase I clinical trial

**DOI:** 10.1038/s41541-021-00342-3

**Published:** 2021-06-02

**Authors:** Leike Li, Daniel C. Freed, Yaping Liu, Fengsheng Li, Diane F. Barrett, Wei Xiong, Xiaohua Ye, Stuart P. Adler, Richard E. Rupp, Dai Wang, Ningyan Zhang, Tong-Ming Fu, Zhiqiang An

**Affiliations:** 1 Texas Therapeutics Institute, Brown Foundation Institute of Molecular Medicine, University of Texas Health Science Center at Houston, Houston, TX USA; 2 Department of Infectious Diseases and Vaccines Research, Merck Research Laboratories, Merck and Co. Inc., Kenilworth, NJ USA; 3 Sealy Institute for Vaccine Sciences, The University of Texas Medical Branch, Galveston, TX USA; 4 Department of Microbiology and Immunology, School of Medicine, Virginia Commonwealth University, Richmond, VA USA

**Keywords:** Live attenuated vaccines, Antibodies

## Abstract

A conditionally replication-defective human cytomegalovirus (HCMV) vaccine, V160, was shown to be safe and immunogenic in a two-part, double-blind, randomized, placebo-controlled phase I clinical trial (NCT01986010). However, the specificities and functional properties of V160-elicited antibodies remain undefined. Here, we characterized 272 monoclonal antibodies (mAbs) isolated from single memory B cells of six V160-vaccinated subjects. The mAbs bind to diverse HCMV antigens, including multiple components of the pentamer, gB, and tegument proteins. The most-potent neutralizing antibodies target the pentamer-UL subunits. The binding sites of the antibodies overlap with those of antibodies responding to natural HCMV infection. The majority of the neutralizing antibodies target the gHgL subunit. The non-neutralizing antibodies predominantly target the gB and pp65 proteins. Sequence analysis indicated that V160 induced a class of gHgL antibodies expressing the HV1-18/KV1-5 germline genes in multiple subjects. This study provides valuable insights into primary targets for anti-HCMV antibodies induced by V160 vaccination.

## Introduction

Human cytomegalovirus (HCMV), a prototypical beta-herpes virus, is a major cause of morbidity and mortality in immunocompromised transplant recipients and congenitally infected fetuses^1,2^. HCMV infection is widespread, with >50% of adults naturally infected worldwide^3^. The majority of primary HCMV infections lack specific clinical symptoms. However, HCMV infection can lead to complications for recipients of organ transplantation^2^. In addition, both primary and secondary infections in women during pregnancy can lead to in utero HCMV infection, which can cause irreversible fetal neurodevelopmental abnormalities with severe consequences such as mental retardation, cerebral palsy, and hearing loss^4^. Each year, an estimated 0.64% of the birth cohort in the United States is born with congenital HCMV infection^5^. Antiviral treatments with nucleoside analogs such as ganciclovir and cidofovir can suppress viral replication and improve the prognosis in immunocompromised patients with HCMV infections^6^. However, they are not of proven value in preventing or treating congenital HCMV infection^7^. The development of an effective vaccine for the prevention of congenital HCMV infection is of great importance.

The HCMV genome encodes at least 19 membrane proteins integral to the virion envelope^8^, which elicit robust immune responses following viral infection. However, the components and the specificities of antibodies that accounted for protection against HCMV infection have not been fully revealed. HCMV infection is a multi-step process typically involving more than one envelope glycoprotein. Thus, the antibodies to a single individual viral antigen are unlikely to be effective against the acquisition and infection of HCMV, which contributes to the challenge of developing the HCMV vaccine^9^. Different HCMV glycoprotein complexes are capable of meditating entry through interaction with cell-specific receptors on a panel of host cells^10,11^. The pentamer (gH/gL/UL128/UL130/UL131A) mediates HCMV entry into epithelial, endothelial, and myeloid cells by its binding to neuropilin 2 (Nrp2)^12^. The trimer (gH/gL/gO) can mediate infection of all cell types by binding to platelet-derived growth factor-alpha (PDGFRα)^13^. Both pentamer and trimer need to interact with the glycoprotein B (gB), the fusogenic protein, to trigger the virus and host cell membrane fusion^14^. Half of the neutralizing activity against HCMV was shown to target gB^15^. However, not only gB but other glycoproteins complex are utilized to gain entry into different cell types, represent important targets for neutralizing antibodies. Indeed, the most-potent HCMV neutralizing antibodies in naturally acquired immunity target the gH-pentamer complex^16,17^.

Natural infection induces potent cellular and humoral immunity in seronegative subjects. The CD4^+^ T-cell response and the pentamer antibody response have been shown to be correlated with a reduced rate of intrauterine HCMV transmission in pregnant women with primary infection^18,19^. In the rhesus monkey model, preexisting antibodies can protect against congenital rhesus CMV infection^20^. Moreover, depletion of maternal CD4^+^ T cells in the monkey delays the neutralizing antibody response and increases viral transmission^21^. Thus, both cellular and humoral responses are likely key components of protective immunity. In addition, HCMV is a highly cell-associated virus^22^. The neutralizing antibodies may not be effective in blocking the cell-to-cell spread of HCMV. The gB non-neutralizing antibodies provide protection by engaging the function of complement-enhanced neutralizing and antibody-dependent cellular phagocytosis^23,24^. Thus, an efficient HCMV vaccine candidate would need to induce both humoral and cellular immune responses to diverse epitopes, requiring a multi-antigen approach.

Several experimental live-attenuated vaccines that utilize laboratory HCMV strains failed to prevent primary infection in clinic trials^25^, and the results may be partially due to the lack of pentamer key for inducing potent neutralizing antibodies^26^. V160 is an experimental vaccine that is genetically modified from AD169 strain with the restored pentamer^27,28^. V160 has been evaluated in phase I clinical trial for safety and immunogenicity in both HCMV-seropositive and seronegative healthy subjects^29^. The vaccine can effectively elicit humoral immune responses qualitatively analogous to those of natural infection as assessed by measuring serum-neutralizing titers longitudinally after vaccination^29,30^. However, the vaccine-induced humoral immune responses have only been analyzed as polyclonal immune sera. This analysis, although effective in measuring the overall inhibition of HCMV infection^30^, would not reveal the antigen specificity of the vaccine-induced antibodies.

To answer this question, we isolated a panel of 272 mAbs from single memory B cells of six subjects. We measured the neutralizing activity as well as binding specificity of these mAbs to pentamer, trimer, gHgL, gB, and tegument proteins. We showed that V160 elicits potent neutralizing antibodies to pentamer, blocking HCMV entry in epithelial cells. V160 also induces neutralizing antibodies to the gHgL, the subunit of trimer and pentamer, able to confer protection of both epithelial and fibroblast cells in vitro. V160 vaccination elicits a large panel of non-neutralizing mAbs targeting gB and pp65. The neutralizing activity of less-potent gB antibodies can be enhanced in the presence of complement. Overall, V160 vaccination in HCMV-seronegative subjects results in a pattern of B-cell responses similar to those of natural infections.

## Results

### Clinical study design

**Fig. 1:**
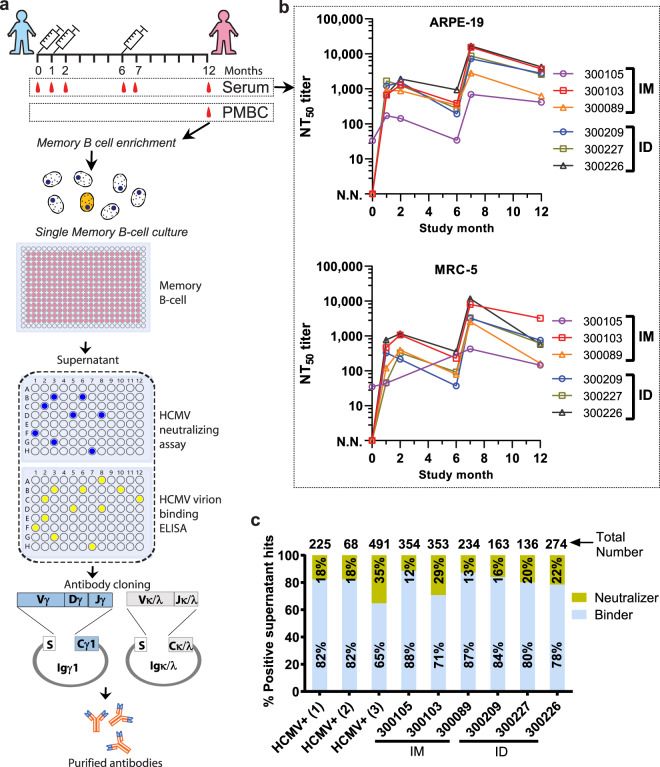
The isolation of V160-elicited monoclonal antibodies from human subjects. **a** Flowchart of monoclonal antibody isolation by single B-cell culturing and antibody cloning. After three-dose vaccination, the memory B cells were isolated from six subjects and cultured in 384-well plates. After 14 days, the single-cell culture supernatants were tested for HCMV neutralization in ARPE-19 cells and HCMV virion binding. Positive supernatants were selected based on the neutralizing and binding activity, and antibody genes were cloned from the positive wells. The antibodies were expressed and purified for binding and neutralization assays, and epitope mapping. **b** Longitudinal neutralizing titers of V160-vaccinated subjects in ARPE-19 and MRC-5 cells. Three subjects (300105, 300103, 300089) were injected intramuscularly (IM), another three subjects (300209, 300227, 300226) were injected intradermally (ID). The serum of the subjects was taken at six time points: month 0, 1, 2, 6, 7, and 12. The reciprocal serum dilution that achieves 50% viral neutralization (NT_50_) was calculated using a four-parameter curve fitting. **c** Functional distribution of neutralizing and binding antibodies in the screening with supernatants of single memory B-cell cultures. The numbers of positive hits for each subject are placed above the columns. The percentages of neutralizers and binders in each subject are labeled in the columns. Three HCMV-seropositive subjects (HCMV+) are positive controls. N.N. indicates no neutralizing activity.

V160 was previously tested in four groups injected intramuscularly (IM) at 10, 30, 100, or 250 U/dose, and one group injected intradermally (ID) at 30 U/dose in a phase I clinical trial, which recruited 190 healthy subjects^29,30^. In this sub-study, to isolated the vaccine-induced monoclonal antibodies, and compare its pattern induced by two routes of administration, we performed the single memory B-cell culturing of three subjects in the 30 U (IM, seronegative) group, and three subjects in 30 U (ID, seronegative) group (recruitment started, November 2013). The V160 vaccine was administered as a three-dose regimen at month 0, month 1, and month 6. The single memory B cells were isolated and cultured at month 12 (Fig. 1a and Supplementary Table 1). Three HCMV-seropositive donors were recruited as the positive controls.

The longitudinal serum-neutralizing titers were evaluated before the antibody isolation. The titers are reported as the reciprocal of serum dilutions needed to achieve inhibition of 50% input virus entry into epithelial ARPE-19 cells (NT_50_) and fibroblast MRC-5 cells (Fig. 1b). The first (month 0) and second (month 1) doses induced neutralizing titers in the range of 20-1,000. The third dose, at month 6, induced a 10-fold increase of that to 5000–10,500. The neutralizing titers in the six subjects gradually declined and remained in the range of 100–10,000 in both ARPE-19 and MRC-5 cells at month 12 (completion time point of the sub-study) (Fig. 1b). In both ARPE-19 and MRC-5 cells, the titers (at month 7) induced by vaccination were in the same range as those elicited by natural infection (Supplementary Table 1). This result indicates the successful establishment of HCMV-specific memory B cells in the vaccinated subjects, necessary for isolation of monoclonal antibodies.

### Isolation of HCMV-specific monoclonal antibodies

We aimed to comprehensively profile the B-cell response by cloning an extensive panel of monoclonal antibodies from the individual peripheral memory B cells of the vaccinated subjects. The whole-virion vaccine contains multiple viral antigens that could trigger different antibody responses^30^. To maximize the diversity and avoid the bias toward enriching higher affinity memory B cells by antigen-specific baiting, we cultured 64,000 single IgG^+^ memory B cells from each subject without antigen selection. The B cells were isolated and cultured for two weeks. We assessed 10 plates from each subject and found an average of 45% IgG-positive wells (Supplementary Table 2), a sign of consequential differentiation of memory B cells to antibody-producing cells. Culture-conditioned B-cell supernatants containing target antibodies are screened in both neutralizing and binding assays (Fig. 1a and Supplementary Table 2). We scored the antibody in the supernatant as a hit if it had HCMV virion binding signal of >10-fold above background (named binder) or >50% reduction of HCMV infection in ARPE-19 cells (named neutralizer).

In each of the six subjects, we identified 136 to 354 supernatants that were positive in either binding or neutralization assays (Supplementary Table 2). The numbers of total, neutralizing, and binding supernatants were comparable for B-cell cultures of vaccinees in the two administration route groups (ID and IM) (Fig. 1c). The majority of the mAbs bind to HCMV virion are non-neutralizing binders, which range from 71% to 88% of the total hits, comparable to the 65% to 82% binders found in the supernatants of B-cell cultures from naturally infected subjects (Fig. 1c). The percentage of neutralizing supernatants ranges from 12% to 29% for B-cell cultures of the subjects, with the lowest of 12% for vaccinee-300105 and the highest of 29% for vaccinee-300103. To elucidate the humoral immune responses to V160 vaccination at monoclonal antibody level. We cloned the paired VH and VL genes from all the neutralizing hits, and a subset of binding hits, which are randomly selected with a number equal to that of neutralizing hits for each subject. Altogether, we isolated 272 mAbs from six subjects by single B-cell culturing (Supplementary Table 3).

### V160 elicits diverse potent antibodies to HCMV antigens

**Fig. 2:**
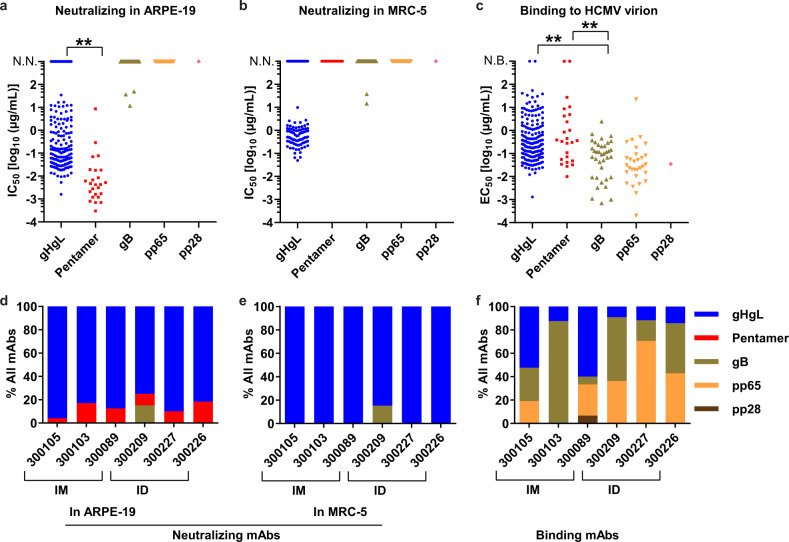
V160 induces neutralizing and binding antibodies targeting diverse antigens in the HCMV-seronegative subjects. Neutralization potency was quantified by IC_50_, defined as the concentration of the IgG required to block 50% viral entry into ARPE-19/MRC-5 cells. The binding affinity of the antibodies to HCMV virion was quantified by EC_50_, defined as the concentration of the IgG required to achieve 50% of the maximal binding signals in ELISA. **a** The neutralization potency in ARPE-19 cells of mAbs grouped based on their antigen specificity. **b** The neutralization potency in MRC-5 cells of mAbs grouped based on their antigen specificity. **c** The relative binding affinity of mAbs based on their antigen specificity. **d** Distribution of neutralizing mAbs targeting different antigens in each of the six subjects as assessed in ARPE-19 cells and **e** MRC-5 cells. **f** Distribution of binding mAbs targeting different antigens in each of the six subjects. The subjects are indicated with the subject numbers 300105, 300103, 300089 in the intramuscular injection group (IM), and 300209, 300227, 300226 in the intradermal injection group (ID). Unpaired two-tailed *t* test was performed. Differences with statistical significance are shown with *p* values, “**” indicates *p* < 0.001. The data are representative of two independent experiments. N.N. indicates no neutralizing activity. N.B. indicates no binding to the HCMV virion.

We determined the antibody-binding specificity to HCMV antigens, including gHgL, pentamer, trimer, and gB, as well as tegument proteins such as pp65, pp28, pp71, and pp150 (Supplementary Fig. 1). We screened antibodies for binding to the antigens by enzyme-linked immunosorbent assay (ELISA). Because both the pentamer and trimer contain gHgL subunit, the antibodies binding to gHgL, pentamer, and trimer simultaneously are defined as gHgL antibodies. Antibodies binding only to pentamer are defined as pentamer-specific antibodies, which bind to pentamer-ULs (UL128/UL103/UL131A), or conformational epitopes composed of gHgL and ULs. Antibodies binding only to trimer are defined as trimer-specific antibodies, which bind to gO or conformational epitopes. The results show that the isolated antibodies bind to gHgL, pentamer, gB, pp65, and pp28, but not to trimer, pp71, and pp150 (Fig. 2 and Supplementary Table 3).

We assessed the antibody-neutralizing activity (IC_50_) in ARPE-19 epithelial cells and MRC-5 fibroblast cells, and their relative binding affinity to HCMV virion (EC_50_) in ELISA (Fig. 2a–c, Supplementary Fig. 2 and Supplementary Table 3). The neutralizing activity of pentamer-specific mAbs was potent, with IC_50_ values ranging from 0.0007 to 8.5 µg/ml in ARPE-19 epithelial cells. Nearly 72% (18 in 25) of the pentamer-specific mAbs inhibits HCMV infection with IC_50_ values in the 0.0007–0.01 µg/ml range (Fig. 2a). As expected, none of the pentamer-specific antibodies can block HCMV infection in fibroblast cell line MRC-5 (Fig. 2b). In contrast, the neutralizing activity of gHgL mAbs is lower than that of pentamer mAbs. The gHgL mAbs had a mean IC_50_ of 0.38 µg/ml in ARPE-19 cells. Only 5 of the 179 gHgL mAbs have IC_50_ values lower than 0.01 µg/ml (Fig. 2a). These results are consistent with the findings that the most-potent neutralizing antibodies target the pentamer in HCMV naturally infected individuals^16^.

V160 induced a large proportion of gHgL mAbs (66%, 179 of 272) in all six subjects (Fig. 2d, e). In the 179 gHgL antibody panel, 152 mAbs inhibit HCMV entry into ARPE-19 epithelial cells (Fig. 2a, d). About 60% of the gHgL-neutralizing antibodies (91 of 152) show activity against viral entry into both ARPE-19 and MRC-5 cells (Fig. 2b, e).

Of the 37 gB-targeting antibodies, only two weakly inhibited HCMV infection in both ARPE-19 and MRC-5 cells (mAb-858: IC_50_ = 37.3 µg/ml in ARPE-19, IC_50_ = 38.1 µg/ml in MRC-5; and mAb-862: IC_50_ = 11.8 µg/ml in ARPE-19, IC_50_ = 14.7 µg/ml in MRC-5) (Fig. 2a, b and Supplementary Table 3). Notably, the gB mAbs on average bind to the HCMV virion with higher affinity than the mAbs against other antigens (Fig. 2c). Of the 18 top binders with EC_50_ values < 0.01 µg/ml, 10 mAbs bind to gB (Fig. 2c). In contrast, gHgL and pentamer antibodies show lower binding to HCMV virion than that of the gB antibodies. The EC_50_ values of gHgL mAbs are above 0.01 µg/ml, with the exception of one mAbs (mAb-323, EC_50_ = 0.0013 µg/ml) (Fig. 2c). Only one pentamer-specific antibody exhibited high-affinity virion binding (mAb-583, EC_50_ = 0.01 µg/ml) (Fig. 2c). These results are consistent with those observed in the antibodies of naturally infected subjects^16^.

A total of 30 pp65 antibodies were isolated from five of the six subjects. One pp28 antibody (mAb-143) was isolated from vaccinee 300089 (Fig. 2c, f and Supplementary Table 3). None of them inhibits HCMV infection in APRE-19 or MRC-5 cells. Notably, 6 of the 30 pp65 mAbs bind to HCMV virion with high affinity at EC_50_ values < 0.01 µg/ml. One antibody mAb-169 has the highest affinity with EC_50_ at 0.0002 µg/ml (Fig. 2c). V160 induces more pp65 mAbs through ID injection (73%, 22 of 30 mAbs) than through IM injection (27%, 8 of 30 mAbs) (Fig. 2f). Overall, the results indicated that V160 vaccination induces diverse and potent neutralizing antibodies to multiple HCMV antigens.

### The pentamer antibodies bind to the protective epitopes

**Fig. 3:**
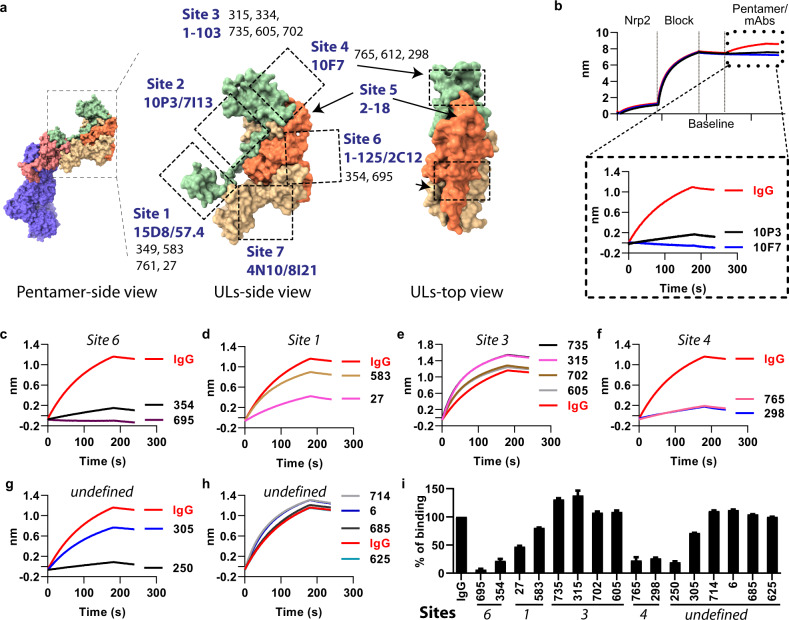
Epitope mapping of pentamer-specific mAbs. The binding sites of pentamer-specific antibodies were mapped by competitive ELISA and biolayer interferometry (BLI) assay. **a** Structure of pentamer (PDB: 5VOD) and antibody-binding sites. Pentamer mAbs bind to seven reported sites (sites 1–7) mapped by reference antibodies (labeled in dark blue). The dashed rectangles and the arrows indicate antibody-binding regions. V160-induced pentamer mAbs (labeled in black) are mapped to four sites on the pentamer which include sites 1, 3, 4, and site 6. **b** Measuring antibody blocking of Nrp2/pentamer interaction by a BLI assay, which measures the light shifts (nm) upon changes in the thickness of the biolayer. The shift depends on the molecular weight and the affinity of the interactors. The Nrp2 protein was loaded on the NTA-biosensor and then incubated with the pentamer/mAb mixture. The positive control mAbs 10P3 and 10F7 inhibit pentamer binding to the Nrp2, but not the IgG control. A subset of pentamer mAbs was tested for blocking of pentamer/Nrp2 interaction as below. **c** Two site 6 mAbs block the Nrp2/pentamer interaction. **d** Two site 1 mAbs partially block the Nrp2/pentamer interaction. **e** Four site 3 mAbs do not block the Nrp2/pentamer interaction, the *Y* axis indicates the shift upon the binding of pentameric-antibody complex, the signal is higher than the pentamer alone (with IgG control). **f** Two site 4 mAbs block the Nrp2/pentamer interaction. **g**, **h** Antibodies with undefined binding sites can be divided into blocking (mAbs-250), partially blocking (mAb-305), and non-blocking (mAbs-714, 6, 685, 625) groups. **i** The percentage of Nrp2/pentamer binding in the presence of pentamer-specific antibodies at different binding sites normalized with the control IgG. Error bars indicate the standard deviation. The Molecular graphic images were produced using UCSF ChimeraX (http://www.cgl.ucsf.edu/chimerax/).

As the most-potent neutralizing antibodies target pentamer, we mapped the antibody-binding sites on the pentamer by pairwise competition ELISA with a panel of well-defined reference antibodies. The binding sites on the pentamer of the reference antibodies were described in two previous studies (Fig. 3a)^31,32^, which include five non-overlapping sites defined by antibodies 10P3/7I13, 4N10/8I21, 2C12, 15D8, and 10F7^31^, and four sites defined by antibodies 1–125, 2–18, 1–103, and 57.4^32^. We first identified the overlapping binding sites of the reference mAbs from the two previous studies. Results show that 1–125 competes for the binding with 2C12, and 57.4 competes with 15D8 (Supplementary Table 4). Based on this result, we consolidated all the binding sites into seven non-overlapping antigenic sites on the pentamer (Fig. 3a). These include UL128-α3 (site 1), the linker of UL128-β6-α3 (site 2), ULs (site 3), UL128/131 A tip (site 4), N-terminal of UL130 (site 5), UL130/131 A β sheet (site 6), and UL128/130/131 A arm (site 7). By performing pairwise competition ELISA, we mapped the V160-induced pentamer mAbs to four antigenic sites: four mAbs (349, 583, 761, 27) bind to site 1, five mAbs (315, 334, 735, 605, 702) bind to site 3, three mAbs (765, 612, 298) bind to site 4, and two mAbs (354, 695) bind to site 6 (Fig. 3a and Supplementary Table 5). It is noted that these four sites are targeted by the potent pentamer-specific neutralizing antibodies isolated from naturally infected subjects^16,33^, the antibody response to the protective epitopes was shown to be associated with a reduced risk of HCMV transmission to the fetus in pregnant women^19^.

Nrp2 is one of the receptors permitting HCMV entry into epithelial cells^12^. To demonstrate whether the V160-induced mAbs inhibit HCMV infection by blocking pentamer-Nrp2 binding, we first loaded the Nrp2 ectodomain protein onto the biolayer interferometry (BLI) biosensor. Then, we incubated the biosensor with the pentamer/mAb mixture. As expected, the positive control mAbs, 10P3 and 10F7, but not the IgG control antibody, inhibited the binding of pentamer to the Nrp2 on the biosensor (Fig. 3b). Testing of V160-induced pentamer mAbs shows that site 6 and site 4 mAbs strongly block the pentamer/Nrp2 interaction to lower than 20% of the IgG control (Fig. 3c, f, i). Site 1 mAbs partially inhibited the interaction to 50% by mAbs-27, and 89% by mAb-583 (Fig. 3d, i), whereas site 3 mAbs do not block the interaction (Fig. 3e, i). Six pentamers neutralizing mAbs without defined binding sites were also included in the receptor blocking assay (Fig. 3g, h). These mAbs could be divided into three categories based on their ability to block pentamer/Nrp2 interaction: strong blockers, such as mAbs-250; intermediate blockers such as mAb-305 that reduces the pentamer/Nrp2 binding by 25%; and non-blockers, which include four mAbs (714, 6, 685, and 625) (Fig. 3g–i).

### The gB antibodies target the antigenic domain 1 with complement-enhanced neutralizing activity

**Fig. 4:**
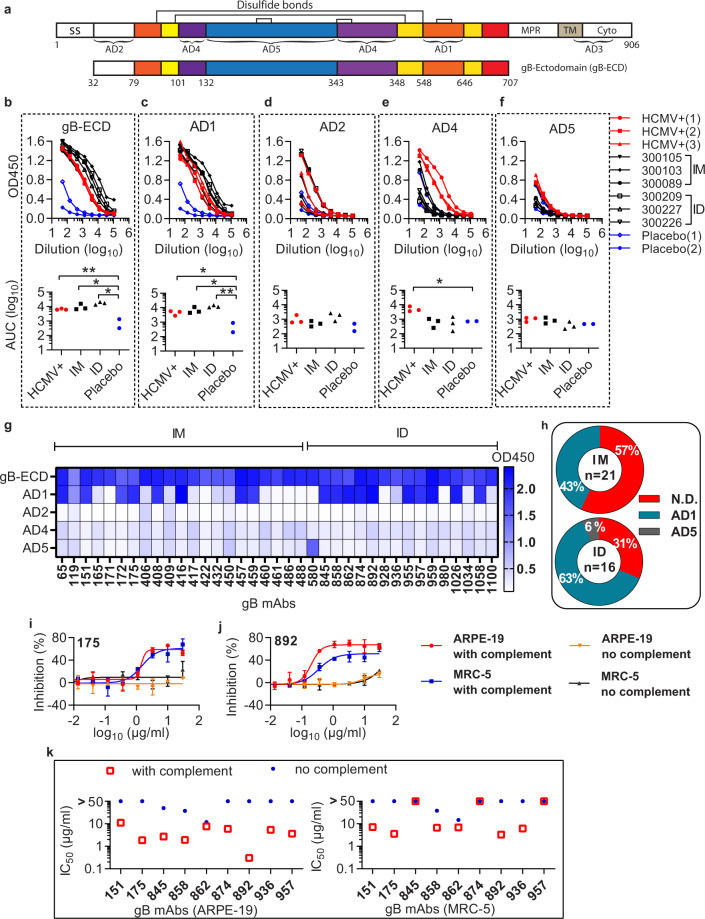
Complement-enhanced neutralizing activity of gB antibodies. **a** Schematic representation of the full-length HCMV gB (top, #ACL51135) and gB-ectodomain (gB-ECD) (bottom). Disulfide bonds are represented as black brackets, antigenic domains (AD1, AD2, AD4, AD5) are indicated in black brackets. Structural domains are labeled as, signal sequence (SS), membrane-proximal region (MPR), transmembrane domain (TM), cytoplasmic domain (Cyto). Numbers denote construct boundaries. **b**–**f** ELISA measuring plasma antibody binding to gB-ECD (**b**), AD1 (**c**), AD2 (**d**), AD4 (**e**), and AD5 (**f**). The top panel shows optical density units at OD450 nm (*Y* axis) and reciprocal plasma dilutions (*X* axis). The sera of HCMV-seropositive subjects (HCMV+), the placebo control (Placebo), and the vaccinated subjects are indicated. The bottom panel shows the normalized area under the curve (AUC) of the ELISA. An unpaired two-tailed *t* test was performed. Differences with statistical significance are shown with *p* values, “**” indicates *p* < 0.001. “*” indicates *p* < 0.05. The data are representative of two independent experiments. **g** Thirty-seven gB mAbs recognize four antigenic domains as determined by ELISA. The recombinant gB-ECD, AD1, AD2, AD4, and AD5 were coated at 3 µg/ml on ELISA plates, and tested for reactivity with gB antibodies. The heatmap indicates the value of OD450 nm of the mAbs to different gB domains in ELISA. **h** Distribution and binding specificity of gB mAbs from the intramuscular injection group (IM) and the intradermal injection group (ID). **i**, **j** The gB mAb (175 and 892) was mixed with AD169r-GFP virus with or without rabbit complement in neutralizing assay. The neutralizing activity is detected by GFP+ cell counting at 44 hours after infection. Error bars indicate the standard deviation. **k** Complement-enhanced neutralizing activity of nine gB antibodies in ARPE-19 and MRC-5 cells. The IC_50_ values defined as antibody concentrations required to neutralize 50% of viral infectivity were calculated by four-parameter curve fitting. The rabbit complement was added at 1:64 final dilution. The results shown are representative of two independent experiments.

The HCMV gB protein has five antigenic domains (AD1 to AD5), which are the binding hotspots of serum antibodies from HCMV-positive subjects (Fig. 4a)^34^. To determine the binding sites of gB antibodies induced by the vaccination, we performed binding titration of the serum of the six subjects to four gB antigenic domains: AD1, AD2, AD4, AD5, and the gB-ectodomain (gB-ECD) (Fig. 4b–f). We calculated the area under the curve (AUC) to quantify the serum response to different gB domains. Two placebo sera and three HCMV-positive sera were included as controls. Both IM and ID injections of V160 elicit robust serum binding to gB-ECD and AD1 domains, which is similar to that in HCMV-positive sera (Fig. 4b, c). The binding signal is 10-fold higher than that of the placebo controls with the serum at 1000 dilution (Fig. 4b, c). In contrast, V160 vaccination did not induce a significant serum response to AD2, AD4, and AD5 domains (Fig. 4d–f). The binding of serum of HCMV-positive subjects is also low to AD2 and AD5 domains, with no significant difference to the placebo control (Fig. 4d, f). However, all three HCMV-positive sera show binding to AD4 domain, at 100–1000 dilution, which is not observed in the vaccinated subjects (Fig. 4e).

To confirm the results, we mapped the purified 37 gB mAbs to the gB antigenic domains by ELISA. As expected, all the mAbs bind to the gB-ECD protein (Fig. 4g). The most frequent binding site is the AD1 domain (51%, 19 of 37). Only one mAb (mAb 580) binds to AD5, whereas no mAbs bind to the AD2 and AD4 domains (Fig. 4g). The remaining 45% (17 of 37) of gB mAbs did not bind to any of the four individual domains, suggesting that these mAbs bind to conformational epitopes across different domains. IM injection yielded more gB mAbs than that of ID injection (IM = 21 vs. ID = 16). However, ID injection elicited a higher percentage of AD1 mAbs than did IM injection (63% vs. 43%) (Fig. 4h).

In a prior study, we observed that complement can enhance the neutralizing activity of gB antibodies^23^. To assess the complement-enhanced neutralizing activity of the gB antibodies, we determined the neutralization IC_50_ values of the mAbs in both ARPE-19 and MRC-5 cells with or without rabbit complement (Fig. 4i–k). Nine gB mAbs show complement-enhanced neutralizing activity in ARPE-19 cells, with the IC_50_ ranging from 0.3 to 11 μg/mL with complement, in contrast to IC_50_ of above 50 μg/mL without complement (Fig. 4k). Two of the nine mAbs (mAb-175, 892) neutralize HCMV infection in APRE-19 cells at the IC_50_ of 1.8 and 0.3 μg/mL with complement, whereas their neutralizing activity is undetectable without complement (Fig. 4i, j). Six of the nine gB mAbs also efficiently inhibit HCMV infection with complement in MRC-5 cells, and two representatives mAb-175 and mAb-892 exhibit the complement-enhanced neutralizing activities at the IC_50_ of 3.3 and 3.6 μg/mL, respectively (Fig. 4i, j). Notably, of the nine gB mAbs with complement-enhanced neutralizing activity, eight mAbs bind to gB-AD1 domain. The other one, mAb-936, binds to an undefined gB epitope (Fig. 4g, k).

### The gHgL antibodies bind to diverse epitopes

**Fig. 5:**
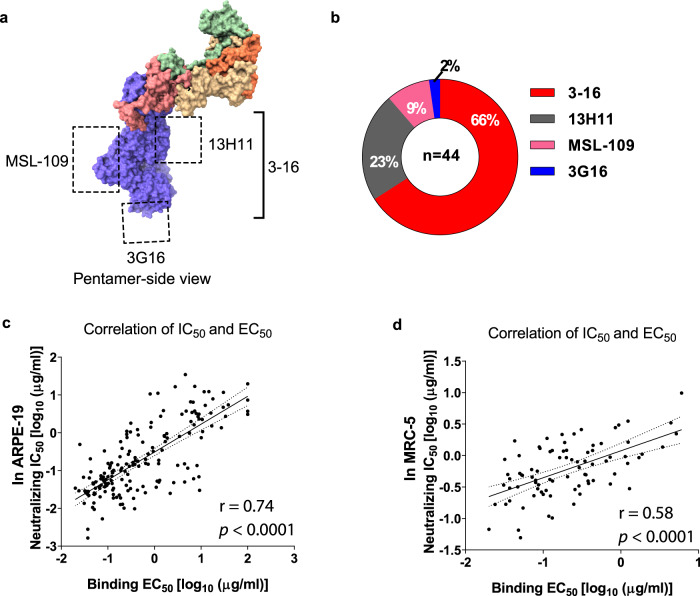
The gHgL mAbs bind to diverse epitopes on the gHgL. **a** The binding sites of four gHgL reference antibodies: 3–16 binds to the gH of pentamer, 13H11 binds to gHgL kinked region, 3G16 binds to the C-terminal domain of gH, MSL-109 binds to the H2 domain of gH as indicated. **b** Forty-four gHgL mAbs bind to overlapping regions with the four reference mAbs: 66% to the 3–16 epitope, 23% to the 13H11 epitope, 9% to MSL-109 epitope, and 2% to the 3G6 epitope. **c**, **d** Neutralization potency (IC_50_) of the gHgL mAbs in ARPE-19 cells (**c**), and MRC-5 cells (**d**), positively correlates with their binding affinity (EC_50_) to the HCMV virion. The IC_50_ and EC_50_ values shown are representative of two independent experiments. The linear association was calculated by the Pearson correlation, two-tailed. The Molecular graphic images of pentamer (PDB: 5VOD) were produced using UCSF ChimeraX (http://www.cgl.ucsf.edu/chimerax/).

The majority of the antibodies induced by V160 target gHgL (66%, 179 of the 272 mAbs) in all six subjects, suggesting that gHgL is the immunodominant antigen of the V160 vaccine. The binding region of four human gHgL reference mAbs has been determined previously: 3–16 binds to the gH of pentamer^32^, 13H11 binds to gHgL kinked region^31^, 3G16 binds to the C-terminal domain of gH^31^, MSL-109 binds to the H2 domain of gH^35^ (Fig. 5a). By competitive ELISA with the reference antibodies, we mapped the binding sites of the gHgL mAbs in this study. Of the 179 V160-induced gHgL mAbs, 44 mAbs bind to overlapping sites with the reference mAbs: 29 mAbs compete with 3–16, 10 mAbs compete with 13H11, 4 mAbs competes with MSL-109, and one mAb-855 competes with 3G16 (Fig. 5b and Supplementary Table 6). These results indicate that V160 vaccination elicits neutralizing mAbs to diverse epitopes on the gHgL glycoprotein complex. It is noted that the binding sites of the four reference mAbs were isolated from HCMV-seropositive subjects, suggesting that V160-induced gHgL targeting mAbs similar to that of natural HCMV infection. It is also noted that a majority (135 of 179) of the gHgL mAbs did not compete with any of the four reference antibodies, suggesting that these mAbs bind to undefined epitopes in gHgL glycoprotein complex. Analysis of the relationship between binding affinity to HCMV virion and neutralization potency demonstrated that most of the potent gHgL-neutralizing mAbs bound to HCMV virion with high affinity (Fig. 5c, d).

### Biased usage of the HV1-18/KV1-5 germline genes in V160-induced gHgL in multiple donors

**Fig. 6:**
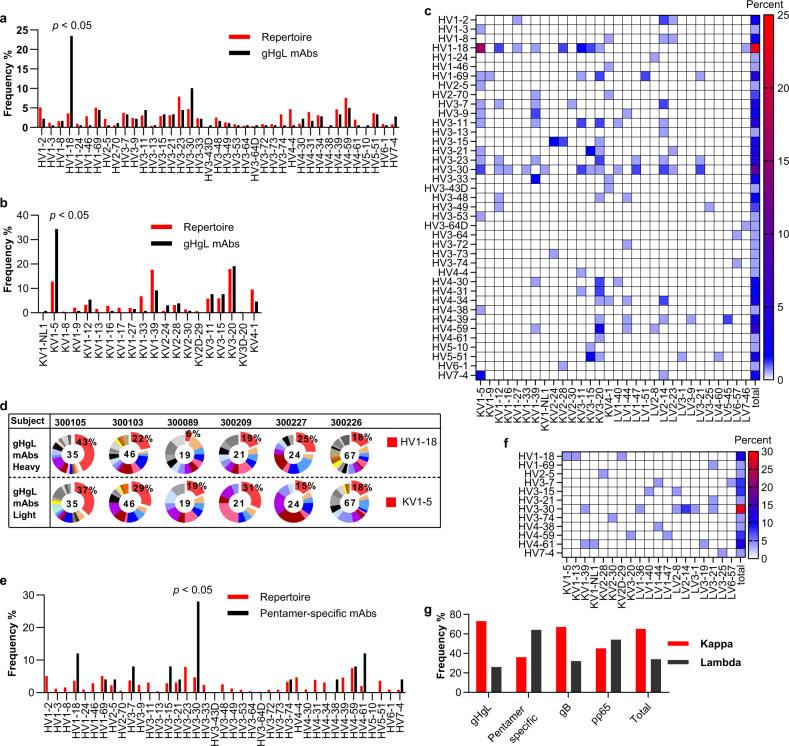
The germline usage of gHgL and pentamer-specific antibodies. **a** Heavy chain variable region germline gene distribution of gHgL mAbs in the six subjects (black bars), compared to the naive variable region gene repertoire (red bars). **b** The kappa chain variable region germline gene distribution of gHgL mAbs in the six subjects (black bars), compared with the naive variable region gene repertoire (red bars). **c** Heatmap frequency of V-gene usage of the paired gHgL antibodies. **d** The pie chart of antibody variable region gene germline distribution in each subject for the gHgL antibodies. Red color indicates the HV1-18 germline, and the KV1-5 germline. The numbers of antibodies of each subject are shown in the middle of the pie. The percentage of HV1-18, and KV1-5 germline are indicated. **e** Heavy chain variable region gene germline distribution of the pentamer-specific mAbs in the six subjects (black bars), compared to the naive variable region gene repertoire (red bars). **f** Heatmap frequency of V-gene usage of the paired antibodies to pentamer. **g** The light-chain pairing preference of mAbs to different HCMV antigens. The reference naive antibody variable region gene repertoire is from a previous study^36^.

Sequence analysis revealed that the antibody repertoires induced by V160 in the six subjects were diverse and each B-cell receptor (BCR) repertoire encompassed the majority of the antibody germline lineages (Supplementary Fig. 3a–c and Supplementary Table 3). By comparing the BCR repertoires of naive subjects^36^, we observed a dominant heavy chain HV1-18 germline and a light-chain KV1-5 germline in V160-induced gHgL antibodies (Fig. 6a, b). This biased germline usage was not observed in gB mAbs and pp65 mAbs (Supplementary Fig. 3d–f). Of the 179 gHgL mAbs, 23% (42 of 179) express the heavy chain HV1-18 germline, 25% (45 of 179) express the light-chain KV1-5 germline (Fig. 6a, b). Remarkably, 16% (29 of 179) of gHgL mAbs express the paired HV1-18/KV1-5 germline (Fig. 6c). The biased germline expression is observed in all but one subject (300089) (Fig. 6d).

When compared with the naive BCR repertoire^36^, a bias towards the heavy chain HV3-30 germline was observed in 28% of the pentamer-specific antibodies, but there was no bias in the light-chain germline genes (Fig. 6e, f). Significantly, a majority (16 in 25) of pentamer-specific antibodies express the lambda light chain, whereas the antibodies to other antigens predominantly express the kappa light chain (Fig. 6g).

## Discussion

An effective HCMV vaccine is urgently needed for the prevention of congenital HCMV infection. Previous experimental vaccines that failed to elicit robust neutralizing antibodies may owing to the lack of pentamer in the attenuated vaccines^37^. The V160 vaccine used in this study is a prophylactic vaccine candidate entering into the clinical trial that is designed with the pentameric complex in its composition^27^. V160 vaccination was effective in priming the immune system and inducing potent neutralizing antibodies in seronegative subjects as assessed by neutralizing assay^29,30^. In this study, we evaluate the breadth and magnitude of V160-induced humoral response at the monoclonal antibody level. We isolated and characterized 272 mAbs and demonstrated that V160 vaccination-induced diverse antibodies to different HCMV antigens in all six seronegative subjects. The activity of the neutralizing antibodies is as potent as that in naturally infected subjects.

V160 vaccination aims at eliciting consequent immunological memory that induces rapid recall responses to HCMV infection. To evaluate the established immune memory, we isolated 64,000 circulating IgG^+^ memory B cells 6 months after the final boost from each of the six subjects. Typically, antigen-specific sorting of the memory B cells is an effective way to enrich the memory B-cell for antibody isolation^38^. However, the pentamer-specific antibodies show a weaker binding affinity to HCMV virion than gB antibodies^16^. To avoid the biased pre-enrichment of memory B cells by antigen baiting, and most importantly to isolate diverse antibodies. We proceeded with the single IgG^+^ memory B-cell culturing without antigen enrichment, then analyzed the binding and neutralizing activity of the supernatant antibodies. About 20% of the supernatant antibodies that bind to the HCMV virion exhibit neutralizing activities, indicating that majority of the antibodies elicited by the V160 vaccine cannot inhibit HCMV infection. Importantly, the frequency of HCMV-specific memory B cells with neutralizing activity in the vaccinated subjects is consistent with that in naturally infected HCMV-seropositive subjects^30^.

In natural infectious, gB, gH-complex, pp65, and pp150 are immunogenic antigens^15,39,40^. The antibodies to these proteins comprise the majority of anti-HCMV antibodies in the serum of seropositive subjects^39,40^. In this study, 75% of the antibodies bind to gH-complex, 14% bind to gB, and 11% bind to pp65. However, we have not isolated all antibodies from the large proportion (71–88%) of single memory B cells that express non-neutralizing antibodies, which mainly bind to gB and pp65 (Fig. 2f). So, V160 may induce a large amount of gB and pp65 antibodies as well as gH-complex antibodies. This is consistent with reports about HCMV antibody targets in naturally infected subjects^16,39^. However, we cannot isolate pp150 antibodies. This may due to the kinetics of antibody response to pp150, which is delayed in primary infection; the antibody titer of pp150 in primary infection is lower than that of non-primary infection^41^. V160 vaccination is not sufficient to induce pp150 antibodies. We also noticed the neutralizing titer in 300105 is 10-fold lower than that in other subjects (Fig. 1). However, the vaccine-induced response in the subject was within the range we reported^30^. In our study, V160-induced potent pentamer antibodies in all six subjects. This was especially notable because the subjects were only exposed to HCMV antigens through three-dose immunizations. The pentamer-specific memory B cells had not passed through the selection of multiple episodes of non-primary HCMV infections common in natural infections. V160 also induced a predominant number of gHgL antibodies, it may correlate with a large number of antigenic sites of the antigen. Similarly, in a prior study, a high proportion of antibodies to gHgL (~60%) was induced by a pentamer vaccine in mice^40^. We were able to readily isolated pentamer and gHgL antibodies from the memory B cells of all the subjects in this study, suggesting that the three-dose vaccination strategy establishes persistent immunological memory B cells to the HCMV antigens. We further demonstrate that some of the pentamer-specific mAbs block pentamer-receptor (Nrp2) interaction, and epitopes of the vaccine-induced pentamer mAbs overlap with that of the mAbs from naturally infected subjects. Notably, it has been reported that antibody response to the pentamer epitopes (sites 1, 2, 4 of this study) was associated with a reduced risk of HCMV transmission to the fetus in pregnant women with primary HCMV infection^19,42^. Other groups of pentamer mAbs do not block Nrp2/pentamer interaction, which is also observed in naturally infected subjects^12^. These results suggest that receptors other than Nrp2 mediate HCMV entry into epithelial cells^43,44^. Presumably, the interaction between the unidentified receptors and pentamer is blocked by the V160-induced pentamer antibodies.

Epitopes for gB mAbs are divided into five antigenic domains (AD), namely AD1–AD5^34,45^. All HCMV infected subjects develop antibodies to AD1 and AD4. Only half of the subjects show reactivity to AD2 and AD5^46^. By assessing the epitope binding specificity of 37 gB mAbs, we observed that V160 exhibits biased induction of AD1 antibodies, with 19 of the gB mAbs binding to AD1, one mAb binding to AD5, and the remaining 17 mAbs only binding to the intact gB-ECD. We have not isolated AD4 mAbs, this epitope specificity difference between the gB mAbs from V160 vaccination and those from HCMV naturally infected subjects may be the result of blockage of the AD4 epitope during V160 vaccine production. Alternatively, it may result from the difference in the antigen stimulation process during vaccination and natural infection. It is also shown that the gB/MF59 vaccine can effectively elicit AD1 antibodies, but not AD4 antibodies in seronegative subjects^47^. All but two of the V160-induced gB mAbs have no neutralizing activity. It is consistent with the previous finding that 90% gB mAbs isolated from HCMV-seropositive subjects do not have neutralizing activity^46^. AD1 is a target of non-neutralizing antibodies^48^, whereas potent gB-neutralizing antibodies bind to the AD2-site 1 domain^49^. The protective capacity of AD2 antibodies has been demonstrated in a clinical trial of the gB/MF59 vaccine. The vaccine induces preexisting AD2 antibodies in seropositive transplant recipients. Higher AD2 antibody level is correlated with protection from viremia of the recipients^50^. However, gB/MF59 does not efficiently elicit AD2 antibodies in seronegative subjects, similar to HCMV natural infection and the V160 vaccination in this study^46,47^. Although gB mAbs show weak neutralizing activity in cell-based assays, a prior study has suggested that gB antibodies play an important role in preventing viral entry of the human trophoblast progenitor cells in vitro^51^. Other non-neutralizing mechanisms such as gB antibody-mediated phagocytosis and complement-enhanced neutralizing activity contribute to the protection against HCMV infection by the gB/MF59 vaccine^23,24^. In our gB mAb panel, eight AD1-specific mAbs and one gB-ECD binding mAb exhibited complement-enhanced neutralizing activity. A recent study has analyzed antibody responses, which are associated with protection in the trial of gB/MF59^52^. It indicates that antibody binding to cell-associated gB was the only humoral immune correlate of protection from CMV acquisition. Further evaluation of the antibodies for the binding to cell-associated gB is needed.

HCMV uses multi-protein to initiate the infection of host cells. The gHgL interacts with gB to form the “core fusion machinery,” which is conserved in all herpesviruses^53^. It was reported that blocking gHgL with antibodies can inhibit HCMV^35^ and EBV infections^54^. A recent study demonstrated that immunization with an HCMV core fusion machinery (gH/gL/gB) elicits strong synergistic neutralizing activities in rabbits^55^. Our data showed that V160 vaccine induces a large number of gHgL-neutralizing antibodies in all six subjects. It suggested that the V160-induced gHgL antibodies may disrupt the “core fusion machinery”, which yielding protections against HCMV infection in broad host cell types. Although, the efficacy of gHgL antibody, and its epitopes, in the protection of HCMV infection in human subjects, has not been demonstrated. Several critical residues within the N-terminal (D–I, D–II) of gH have been identified as hotspots for the binding of neutralizing antibodies, such as mAb 13H11 to HCMV^31^ and mAb AMMO1 to EBV^56^. The conformational changes across D–I and D–II might be required for triggering the gHgL interaction with gB in EBV infection^57^. Thus, blocking of the conformation changes by antibodies may serve as one common mechanism of gHgL neutralization. In this study, using the epitope-defined reference antibodies isolated from HCMV-seropositive subjects, we mapped 10 mAbs bind to the overlapped site on D–II with 13H11. It is suggested that the V160-induced antibodies may block the interaction of gHgL with gB. Another five mAbs bind to the gHgL:gHgL dimerization interface, which is the binding site of reference mAb MSL-109^35^. It suggests that a subgroup of V160-induced gHgL antibodies to inhibit HCMV infection by perturbing gHgL:gHgL dimerization. This is the same neutralizing mechanism for MSL-109 which is being tested in clinical trials as prophylactic and therapeutic agent for HCMV infection^35^. It indicated that V160-induced gHgL antibodies may neutralizing HCMV infection through different mechanisms based on the binding site. In the gHgL mAb panel, 33 mAbs overlapped with the reference mAb 3–16. We have demonstrated that 3–16 neutralize HCMV infection in primary placental cells and differentiating cytotrophoblast cells^58^. Similarly, in a mouse model, the gH antibodies induced by a pentamer vaccine, also prevent HCMV infection of human placental cytotrophoblasts^59^. It indicated that V160-induced gHgL mAbs may prohibit HCMV transplacental transmission in women who seroconverted in early gestation. However, both studies show the inhibition potency of gHgL antibody is 100-fold lower than that of pentamer antibodies^58,59^. The efficacy of gHgL antibodies needs to be clarified by further study.

Even with only three subjects in each of the IM and ID injection groups, we observed differences in antigen specificity of the V160-induced antibodies between the two administration routes. The ID injection route tends to induce more non-neutralizing pp65 and gB-AD1 antibodies when compared with the IM injection route. These observations need to be confirmed by further studies with more subjects and antibodies. It is worth noting that we did not isolate any gO-specific mAb in the study. We performed the initial screening using APRE-19 epithelial cells, but not MRC-5 fibroblast cells. The biased screening may have lead to the loss of gO-specific antibodies.

In this study, a large number of gHgL mAbs isolated from six subjects express the HV1-18/KV1-5 germline combination. This result suggests that V160 biasedly induced gHgL antibodies to express unique germlines. A previous study demonstrated that three highly potent gB-AD2 antibodies, derived from different human subjects, express the same pair of V-genes (IGHV3-30 and IGKV3-11)^60^. A structural study of these antibodies indicated that the V-germline-encoded residues (CDR1 or CDR2) contact the antigen, contributing to high affinity^60^. Presumably, protective antibodies are selected and frequently expressed in the immunity against HCMV infection. This may due to the co-evolution of HCMV and humans. A similar finding has been reported in anti-influenza antibody repertoires^38^. However, the germline usage of gHgL antibody in the seropositive subject has not been fully studied. Further comparative studies on the expansion of B cells following HCMV vaccination and natural infection are warranted. Notably, the pentamer-specific mAbs also prefer to express the HV3-30 heavy chain germline, but the light-chain germline is not restricted. This is in contrast with what has not been observed in pentamer mAbs from HCMV naturally infected subjects. About 75% of the pentamer antibodies express the lambda light chain, a similarly observed for pentamer antibodies in naturally infected subjects^16^. The biased usage of lambda light chain has also been reported in antibodies with potent neutralizing activities to HIV infections^61^. A previous study indicates that the paratopes of lambda chain CDRs are more negatively charged than that of kappa chain^62^, and the distinct profiles of kappa and lambda light chains may serve important functions in antigen binding.

In summary, the detailed characterization of 272 monoclonal antibodies from six subjects in this study revealed several properties of V160-induced humoral responses. First, the vaccination was effective in inducing potent neutralizing antibodies to the pentamer in all six subjects, which is consistent with the observation that the pentamer is the target for potent neutralizing antibodies in naturally infected subjects^16^. Second, V160 elicits a large number of gHgL antibodies with moderate potency in preventing HCMV entry in both ARPE-19 and MRC-5 cells. Third, V160 induces gB-specific antibodies have high-affinity binding to virions, but with minimal neutralizing activity against HCMV infection. Nine of the 37 gB-specific antibodies have complement-enhanced neutralizing activity. This result is consistent with the observation of low neutralizing activity associated with the gB/MF59 vaccine-induced mAbs^23,24^. At last, V160 elicits 30 pp65-specific mAbs and one pp28-specific mAb without neutralizing activity. Taken together, the results of this study demonstrated that V160 was effective in inducing robust host antibody response. Whether these assumptions translate into the induction of protective anti-HCMV immunity in humans can only be clarified by moving the vaccine candidates forward to clinical trials.

## Materials and methods

### Study subjects

This study aimed to isolate and analyze the specificity and function of the HCMV vaccine (V160) elicited monoclonal antibodies. It is a sub-study of a previous phase I clinical trial (NCT01986010, registered on 18 November 2013). The trial was a two-part, single-blind, randomized, placebo-controlled, dose-escalating study, conducted at nine clinical sites in the United States. The detailed trial design and the results have been described and published in a separate research article^29^. The trial was performed in conformance with standards of Good Clinical Practice. The protocol was reviewed and approved by the Western Institutional Review Board, Inc., and the Institutional Review Board of the University of Texas Medical Branch. Subjects provided written informed consent before participation. The whole clinical trial recruited 95 HCMV-seropositive and 95 seronegative adult subjects^29^, Part 1 of the study evaluated immunogenicity and safety of 4 different dose levels (10, 30, 100, and 250 U/dose) of V160 formulated with or without aluminum phosphate adjuvant, when administered IM as a three-dose regimen at day 1 (month 0), month 1, and month 6 in HCMV-seropositive subjects and HCMV-seronegative subjects. Part 2 of the study evaluated a three-dose regimen of a medium dose (30 U) of V160 or placebo administered ID using the same dosing schedule in HCMV-seropositive and HCMV-seronegative subjects. Parts 1 and 2 were conducted in parallel.

In this sub-study, to characterize the vaccine-induced monoclonal antibodies, we selected, at random, six vaccinated participants from the 30 U IM injection group (*n* = 3, seronegative, without adjuvant, enrolled in Virginia Commonwealth University), the 30 U ID injection group (*n* = 3, seronegative, without adjuvant, enrolled in University of Texas Medical Branch), and the two placebo controls of the ID injection group (*n* = 2, without adjuvant, enrolled in University of Texas Medical Branch) (Supplementary Table 1). We also enrolled three HCMV-seropositive subjects for positive control (enrolled in University of Texas Medical Branch). Healthy subjects eligible for inclusion in this sub-study were over 18 years of age with body weight >50 kg and body mass index 19–32 kg/m^2^. The pre-specified primary outcome was assessed by evaluating the serum-neutralizing titer at 1 month post the final dose (month 7), the 95% confidence intervals of neutralizing antibody geometric mean titers were calculated^29^. In addition, the longitudinal neutralizing serum titers were evaluated to further characterize the antibody responses at months 0, 1, 2, 6, 7, and 12. The evaluation of the objectives was not changed after trial commencement. Further details on the random allocation sequence, subject disposition, blinding, and adverse event following immunization are available in the published articles of the clinical trial^29^. The trial information can be obtained from Clinical Trial Registration (https://clinicaltrials.gov/ct2/show/NCT01986010).

### Cell lines, viruses, and vaccine

Human ARPE-19 (CRL-2302) retinal pigmented epithelial cells, MRC-5 (CCL-171) embryonic lung fibroblasts cells were obtained from American Type Culture Collection (ATCC) and were maintained according to the supplier’s recommendations^63^. AD169 (VR-538) was obtained from ATCC and propagated in MRC-5 cells. AD169r-GFP virus was a generous gift of Thomas Shenk of Princeton University, and propagated in ARPE-19 cells. The live-attenuated HCMV virus vaccine, V160, was generated by restoring the wild-type pentamer in the AD169 strain as reported^27^. Viruses were produced in MRC-5 or ARPE-19 cells, and viral particles were harvested and purified by centrifugation at 55,000 × *g* for 90 min through 20% sorbitol cushion in 50 mM tris (pH 7.2) and 1.0 mM MgCl_2_. The pellet was resuspended and stored at −80 °C.

### Protein expression and purification

The gHgL dimmer, and the pentameric complex, consisting of gH, gL, pUL128, pUL130, and pUL131A of the AD169 strain, were produced by transient transfection in Expi293F cells with the respective plasmids. The gH open reading frame was modified with a truncation of its cytoplasmic and transmembrane domains for the production of the secreted complex as previously described^64^. The gO expression construct was designed based on the sequence of AD169. The recombinant trimeric complex, consisting of gH, gL, gO of the AD169 strain, was expressed by co-transfection of the three plasmids in Expi293F cells. The recombinant HCMV gB-ectodomain (AA1-707) was designed based on the AD169 strain sequence with furin-cleavage site mutated, several amino acids mutated to increase the solubility, transmembrane domain deleted, and hydrophobic membrane-proximal region deleted as described previously^34^. The gB antigenic domains: AD1 (AA550-636), AD2 (AA22-80), AD4 (AA112-133 + 343-438), AD5 (AA134-343) from the AD169 strain were tagged with C-terminal His tag and expressed in Expi293F cells as described previously^50^. The tegument proteins pp65 and pp71 were cloned into pET22b(+) vector with C-terminal His tag and expressed in BL21(DE3) *E. coli*, and purified according to the manufacturer’s instruction (Novagen, Madison, WI). The pp28 (catalog number: CMV-212) and pp150 (catalog number: CMV-216) proteins were purchased from ProSpec-Tany TechnoGene Ltd. The recombinant Nrp2 (AA23-855) was expressed with C-terminal His tag and N-terminal heavy chain signal peptide (MEWSWVFLFFLSVTTGVHS) in Expi293F cells. Protein expression in Expi293F cells was performed according to the manufacturer’s instructions (Thermo Fisher Scientific). Supernatants of the Expi293F cells were harvested for protein purification using the Ni-NTA Agarose crude column (Invitrogen) according to the manufacturer’s instructions. The GeneBank accession numbers of the proteins are: gH (ACL51135), gL (ACL51176), UL128 (ACL51185), UL130 (ACL51186), and UL131A (AKI26612), gB (ACL51135), pp65 (ACL51152), pp71 (ACL51151), and Nrp2 (NP_003863).

The purity of the proteins was detected by reducing and non-reducing sodium dodecyl sulfate-polyacrylamide gel electrophoresis using 10% precast polyacrylamide gel (Bio-Rad). Samples (4 µg/each line) were mixed with Laemmli sample buffer, and reduced samples contained dithiothreitol at a final concentration of 100 mM. The samples were heated at 95 °C for 10 min and then electrophoresed for analysis as reported in ref. ^64^.

### Memory B culture and supernatant screening

Human memory B cells culture and screening of HCMV-specific antibodies were performed as reported^16,30^. In brief, IgG^+^ memory B cells were enriched using an EasySep memory B Cell kit (StemCell) and seeded at an average of 1.4 B cells/well in 384-well plates with gamma-irradiated feeder cells expressing human CD40L in complete Roswell Park Memorial Institute 1640 medium supplemented with human interleukin-21 (50 ng/ml) (Invitrogen). A total of ~200 plates for each subject were cultured for 14 days in the incubators at 37 °C, with 5% CO_2_ and 93% humidity. B-cell culture supernatants were then collected and tested for HCMV virion binding and neutralizing activities in ARPE-19 cells.

The ELISA binding and neutralization assay were performed with the protocol previously reported^30^. In brief, for binding activity, purified HCMV virion (V160) was used as the antigen and immobilized at 2 μg/ml in PBS on 384-well high-bind plates. The wells with 10-fold higher optical density value at 450 nm (OD450) than that in the negative control were scored as binding positive hits. For neutralization assay, 15 μl of supernatants were mixed with 15 μl of AD169r-GFP virus (~800 pfu) and incubated at room temperature for 1 h. The mixture was then transferred into 384-well plates that were pre-seeded with ARPE-19 cells at 3000 cells per well. The infected ARPE-19 cells were further cultured at 37 °C, with 5% CO_2_ and 93% humidity for 44 h and then imaged on Acumen eX3 microplate cytometer (TTP Lab Tech Ltd, United Kingdom) for GFP-positive cell count. The wells with >50% reduction in GFP expression cell count were scored as neutralization positive hits.

### Antibody cloning and purification

We clone the antibodies from all the neutralizing hits, and only randomly clone ~30% of the binding hits, due to a large number of positive hits in the supernatant screening. Total RNA from the cells in the positive wells was isolated using RNeasy Micro Kit (Qiagen) and converted to cDNA using iScript™ cDNA Synthesis Kit (Bio-Rad). The IgG genes were recovered by PCR using primers^16^ and the detailed methods^65^ that have been described previously. Recombinant antibodies were expressed by transient transfection in Expi293F cells and purified by protein A affinity chromatography as reported in ref. ^65^.

### Direct ELISA

Endpoint dilution titers of each serum sample to recombinant gB-ECD, and truncated gB proteins were determined by direct ELISA. The antigens were separately immobilized at 2 μg/ml in PBS on 96-well microtiter plates (Corning) at 4°C overnight. Plates were washed once with PBST (PBS/0.05% Tween-20 (Sigma)) and then blocked with 1% (v/v) BSA in PBST for 1 h. Human sera were diluted in serial threefold titrations starting from a 1:50 dilution in PBST (with 1% BSA), and then incubated with the antigen-coated plates for 1.5 h. After incubation and washing three times, the horseradish peroxidase (HRP)-conjugated goat Anti-Human IgG F(ab’)_2_ (Jackson ImmunoResearch) was added with the dilution of 1/5000 in PBST for 1 h. After being washed three times with PBST, the plates were then incubated with 100 μl/well of the tetramethylbenzidine (TMB) substrate (Thermo Fisher Scientific), for 3–5 minutes at room temperature. The reaction was stopped by the addition of 50 μl/well of 1 M H_2_SO_4_, and the absorbance was measured at 450 nm on a microplate reader (Molecular Devices). The average of the signal was used for calculating the AUC using GraphPad Prism 8.

Direct ELISA assays were used to evaluate the antibody binding to HCMV gHgL, trimer, pentamer, gB-ECD, truncated gB variants, pp65, pp28, pp71, and pp150. The 96-well microtiter plates (Corning) were coated with 2 μg/ml individual proteins in 50 μl at 4 °C overnight. The ELISA assays were performed with the method described above.

### Biotinylation of the reference antibodies

Sequences of the reference antibodies (10P3, 8I21, 4N10, 7I13, 2C12, 15D8, 10F7, 3G16, 13H11, 3–16, and MSL-109) were obtained from prior studies^32,33,35^. The synthesized antibody gene fragments were inserted into human IgG1 constructs for expression. The constructs of the other reference antibodies (1–125, 2–18, 1–103, 57.4) were stored in our laboratory as described in previous studies^16^. Biotinylation of the reference antibodies was performed using the EZ-Link™Sulfo-NHS-LC biotinylation Kit (Thermo Fisher Scientific), following the manufacturer’s instructions using a 20-fold molar excess of NHS ester-activated biotin to the antibodies.

### Pairwise antibody competition by ELISA

The 96-well microtiter plates (Corning) were coated with 3 μg/ml pentamer or gHgL proteins in 50 μl PBS at 4 °C overnight. Plates were washed once with PBST (PBS/0.05% Tween-20 (Sigma)) and then blocked with 1% (v/v) BSA in PBST for 1 hr. The V160-induced antibodies were used as primary antibodies, which are diluted at the final concentration of 3 μg/ml in 100 μl PBST, and then added into the ELISA plates for 1 h. The biotinylated reference mAbs were used as secondary competitive antibodies at the final concentration of 3 μg/ml in 100 μl PBST and incubated for 1 h. The Streptavidin-HRP conjugate reagents (R&D, diluted 1:200 in PBST, 100 µl/well) were then added. After further incubation for 1 h at room temperature, TMB substrate solution was added to develop the plates (100 µl/well) and the color reaction was blocked after 5 min incubation by addition of 1 M H_2_SO_4_ (50 μl/well). Absorbance values at 450 nm were determined on a microplate reader (Molecular Devices). After each incubation step, plates were washed three times with PBST to remove the unbound antibody. The percent of binding inhibition by the primary mAbs, was calculated based on the reduction of signals with the primary mAb vs. no primary mAbs. The inhibition rate equals: 100%-(OD450 value with the presence of primary mAb/OD450 value without primary mAb)%.

### Neutralization assays

The viral neutralization assay has been described previously^30^. MRC-5 or ARPE-19 cells were seeded on day 1 at 1.2 × 10^4^ or 1.7 × 10^4^ cells/well, respectively, in 50 μl medium per well in 96-well plates. The antibody samples, or the heat-inactivated serum samples, in twofold serial dilutions, were mixed with 6 × 10^4^ pfu/ml of AD169r-GFP virus in equal volume. The mixture was incubated at 37 °C for 1 h and then 50 μl were transferred to MRC-5 or ARPE-19 cells. In the complement-enhanced neutralizing activity assay of gB mAbs, the virus was incubated with the mAbs in the presence or absence of rabbit complement (Cedarlane, Canada) at the final 1:64 dilution. The plates were cultured for 44 h and cells expressing GFP as a surrogate for HCMV infection were then enumerated using Acumen eX3 laser-scanning fluorescence microplate Cytometer (TTP Lab Tech Ltd, United Kingdom). The IC_50_ values were defined as the concentration of mAb, which achieves a 50% reduction in the number of virus-infected cells. The NT_50_ titers were defined as the reciprocal serum dilutions that achieved 50% reduction of viral infection. Both IC_50_ and NT_50_ values were calculated by four-parameter non-linear curve fittings using GraphPad Prism 8.

### BLI assay

To detect pentamer antibody inhibition of the pentamer-Nrp2 interaction, a competition assay was performed on an Octet RED96 using NTA Biosensors (FortéBio). The Nrp2-His protein was diluted to 35 µg/ml in PBST and placed into 96-well microplates. All biosensors were coated with the diluted Nrp2-His for 300 s, and then washed in PBS for 60 s. The biosensors were then incubated with 200 µg/ml His-tagged blocking protein for 300 s to achieve saturation, then washed in PBS for 60 s. Pentamer/antibody mixture was incubated with the biosensors for 300 s with the final concentration of 10 µg/ml for pentamer, and 50 µg/ml for antibody. The signal was recorded for binding of the pentamer to the coated Nrp2-His. The decrease of pentamer association in the presence of antibody was normalized by total binding in the absence of antibody (PBS control) to calculate the percentage of binding inhibition.

### Statistical analysis

All data were graphed and analyzed using GraphPad Prism version 8 (San Diego, CA). The NT_50_, IC_50_, and EC_50_ values were calculated by a four-parameter curve fitting algorithm. The comparison of IC_50_, EC_50,_ and the serum titer between different panels was conducted using an unpaired, two-tailed *t* test, *p* < 0.05 was considered statistically significant. The linear association between the IC_50_ and EC_50_ values of gHgL mAbs was calculated by the Pearson correlation, two-tailed, *p* < 0.05 was considered statistically significant (95% confidence interval).

### Reporting summary

Further information on research design is available in the Nature Research Reporting Summary linked to this article.

## Supplementary information

Supplementary Information

Supplementary Data

Reporting Summary

## Data Availability

All data generated or analyzed during this study are included in this article (and its supplementary files). The detailed study design of phase I clinical trial was published previously^29^. The full trial protocol can be accessed by submitting an enquiry to Merck & Co. via the website: http://engagezone.msd.com/doc/ProcedureAccessClinicalTrialData.pdf.
