# Supplementary material for: A conditionally replication-defective cytomegalovirus vaccine elicits potent and diverse functional monoclonal antibodies in a phase I clinical trial
Source: NPJ Vaccines. 2021 Jun 2;6:79. doi: 10.1038/s41541-021-00342-3 (PMC8172929; doi:10.1038/s41541-021-00342-3)
Supplement: Supplementary file 1 — Supplementary Information [file 41541_2021_342_MOESM1_ESM.pdf]

Supplementary file

A conditionally replication-defective cytomegalovirus vaccine elicits potent and diverse functional monoclonal antibodies in a phase I clinical trial

Leike Li[1], Daniel C. Freed[2], Yaping Liu[2], Fengsheng Li[2], Diane F. Barrett[3], Wei Xiong[1], Xiaohua Ye[1], Stuart P. Adler[4], Richard E. Rupp[3], Dai Wang[2], Ningyan Zhang[1*], Tong-Ming Fu[1,2*], Zhiqiang An[1*]

[1]Texas Therapeutics Institute, Brown Foundation Institute of Molecular Medicine, University of Texas Health Science Center at Houston, Houston, TX 77030, USA
[2]Department of Infectious Diseases and Vaccines Research, Merck Research Laboratories, Merck and Co. Inc., Kenilworth, NJ 07033, USA
[3]Sealy Institute for Vaccine Sciences, The University of Texas Medical Branch, Galveston, TX 77555, USA
[4]Department of Microbiology and Immunology, School of Medicine, Virginia Commonwealth University, Richmond, VA 23298, USA

[*] Ningyan Zhang, Tong-Ming Fu, Zhiqiang An
Email: ningyan.zhang@uth.tmc.edu; tong-ming.fu@uth.tmc.edu; zhiqiang.an@uth.tmc.edu

Contents

Supplementary Figure 1

Supplementary Figure 2

Supplementary Figure 3

Supplementary Table 1

Supplementary Table 2

Supplementary Table 3

Supplementary Table 4

Supplementary Table 5

Supplementary Table 6

Supplementary Dataset

Reference

Supplementary Figures

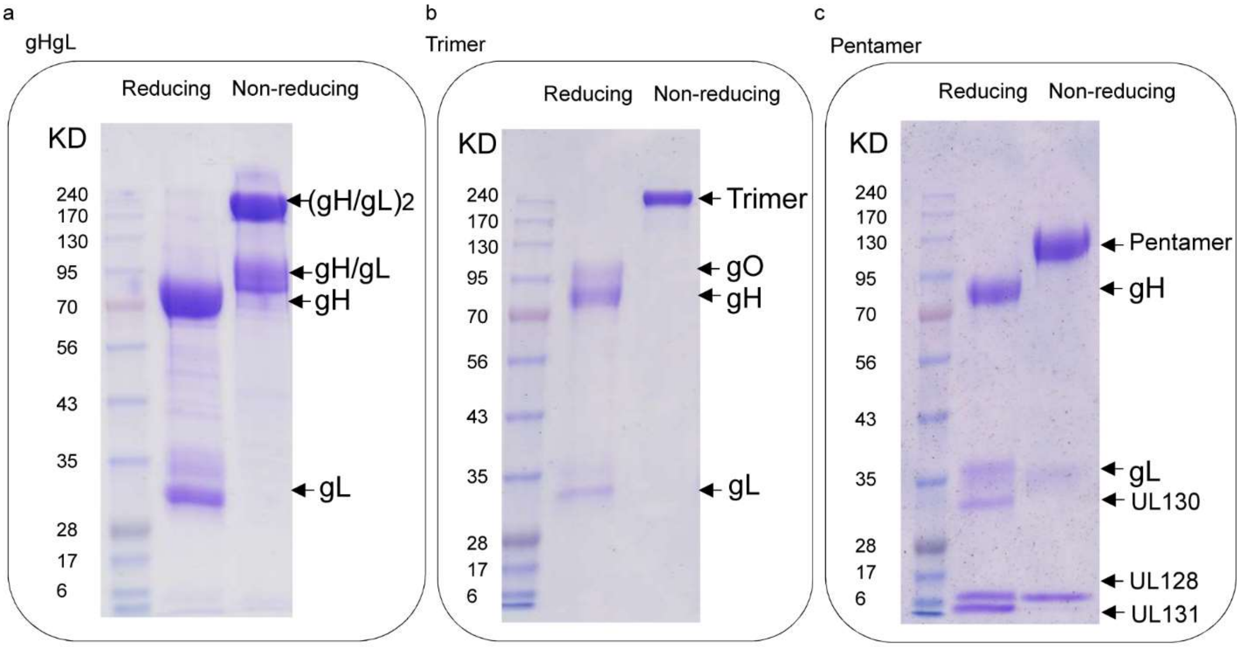

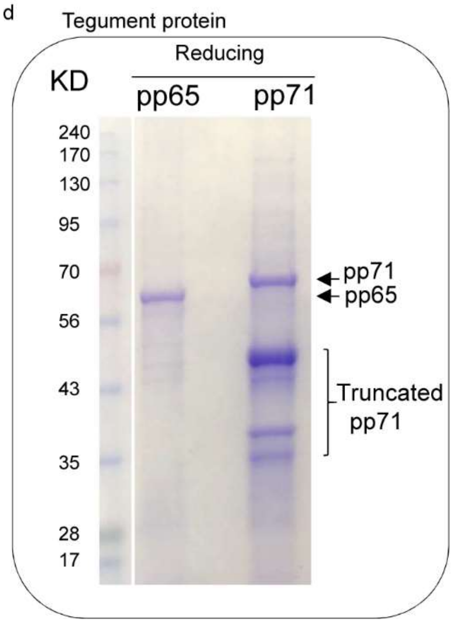

e

Proteins	Targets	Source
Glycoprotein	gHgL	In house purified
	Pentamer	In house purified
	Trimer	In house purified
	gB	Sino Biological
Tegument proteins	pp65	In house purified
	pp71	In house purified
	pp28	ProSpec
	pp150	ProSpec

Supplementary Fig. 1. Expression and purification of HCMV glycoproteins and tegument proteins. The SDS-PAGE of purified HCMV antigens. HCMV glycoproteins gHgL, trimer, and pentamer were expressed in Expi293F cells, and tegument proteins pp65 and PP71 were expressed in *E. coli* BL21. The SDS-PAGE assay was performed in either reducing or non-reducing conditions. **a** HCMV gHgL and gHgL dimer. **b** HCMV trimer. **c** HCMV pentamer. **d** HCMV tegument proteins pp65 and pp71. **e** The list of the antigens used in the study and three

antigens (gB, pp28, and pp150) were purchased from the indicated vendors. The gel pictures were derived from the same experiment and were precessed in parallel.

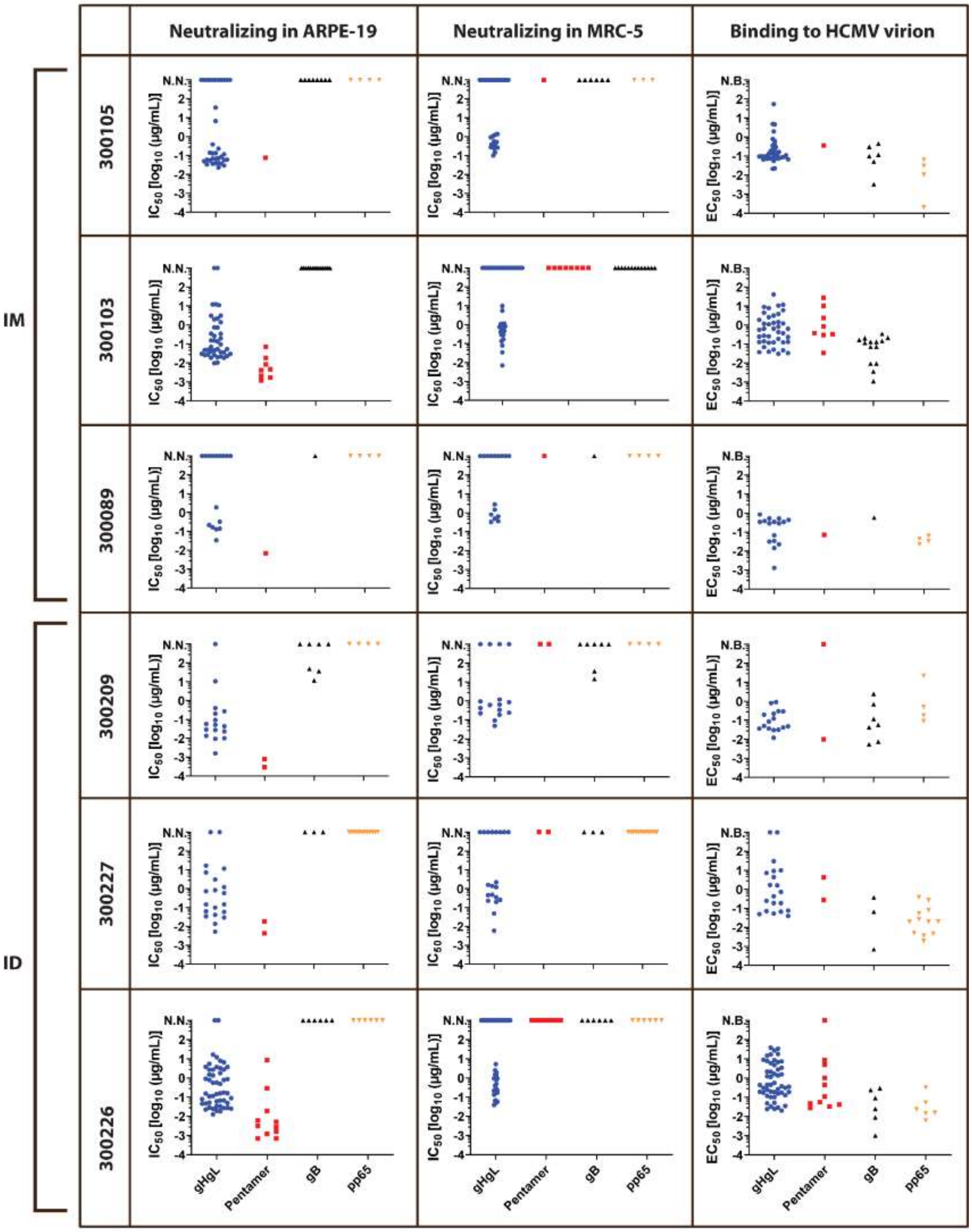

Supplementary Fig. 2. Functional attributes of the HCMV antibodies based on the antigen specificity in each subject. The IC_{50} of mAbs were grouped based on their antigen specificity for their neutralizing potency in ARPE-19 cells (left column) and MRC-5 (middle column) as indicated. The binding affinity of the antibodies to HCMV virion (EC_{50}) (right column). The IC_{50} of antibodies is defined as the concentration of the IgG required to block 50% viral entry into ARPE-19 or MRC-5 cells. The EC_{50} of antibodies is defined as the concentration of the IgG required to achieve 50% of the maximal binding signals in virus-specific ELISA. N.N. indicates no neutralizing activity. N.B. indicates no binding to the HCMV virion. IM, intramuscular injection; ID, intradermal injection.

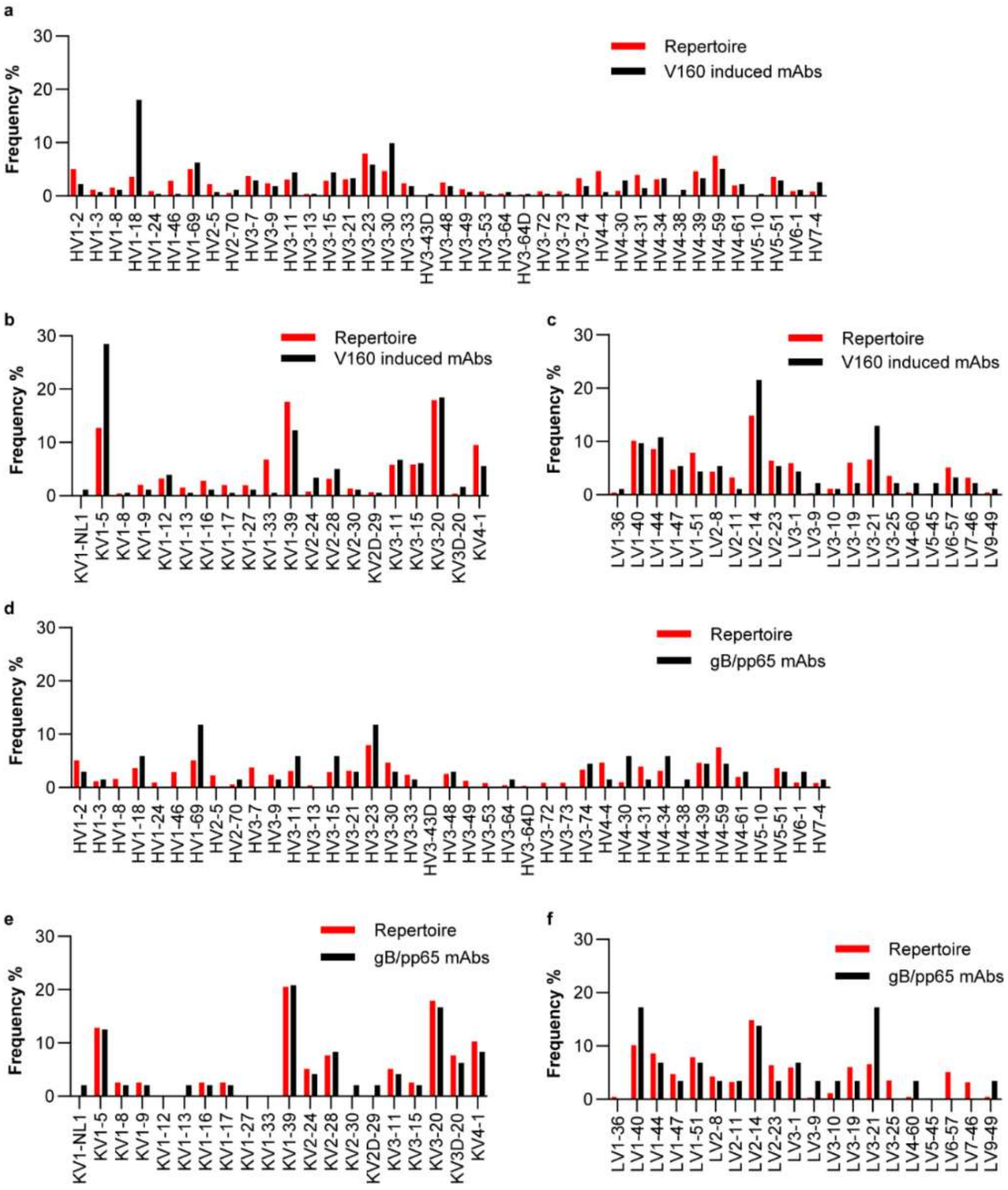

Supplementary Fig. 3. The germline usage of V160 induced antibodies

The black bars indicate the antibodies isolated in this study. The red bars indicate the naive human antibody repertoire from previous study [1]. **a** The heavy chain germline usage of V160 induced mAb. **b** The kappa chain germline usage of V160 induced mAb. **c** The lambda chain germline usage of V160 induced mAb. **d** The heavy chain germline usage of gB and pp65 mAb. **e** The kappa chain germline usage of gB and pp65 mAb. **f** The lambda chain germline usage of gB and pp65 mAb.

Supplementary Tables

Supplementary Table 1. Subject profiles

	Vaccinated subjects						HCMV sero-positive subjects		
Subject	300105	300103	300089	300209	300227	300226	HCMV+ (1)	HCMV+ (2)	HCMV+ (3)
Sex[1]	M	M	F	F	M	M	n/a	n/a	n/a
Age	40s	20s	20s	20s	20s	20s	n/a	n/a	n/a
Administration route[2]	IM	IM	IM	ID	ID	ID	n/a	n/a	n/a
Baseline serum titer[3], In ARPE-19	33	N.N.	N.N.	N.N.	N.N.	N.N.	n/a	n/a	n/a
Baseline serum titer[3], In MRC-5	35	N.N.	N.N.	N.N.	N.N.	N.N.	n/a	n/a	n/a
Serum titer[4], In ARPE-19	695	15474	2844	7260	8693	16726	5562	7406	20007
Serum titer[4], In MRC-5	425	8044	2552	3224	3500	11713	1158	2837	3572

[1]M, male; F, female;
[2]IM, intramuscular injection; ID, intradermal injection;
[3]titer at months 0 for the vaccinated subjects; N.N. indicates no neutralizing activity
[4]titer at months 7 for the vaccinated subjects. For sero-positive subjects, the titers were detected at the time of enrollment.
n/a, not applicable.

Supplementary Table 2. Summary of single memory B cell culturing and antibody cloning

	Vaccinated subjects								
Subject	300105	300103	300089	300209	300227	300226	HCMV+ (1)	HCMV+ (2)	HCMV+ (3)
Number of culture wells	64,000	64,000	64,000	64,000	64,000	64,000	64,000	n/a[1]	64,000
Estimated IgG positive wells	46.8%	57.4%	50.7%	42.9%	42.3%	59.7%	19.2%	32.6%	30.0%
Memory B cell culture supernatant hits									
Neutralizing supernatants	41	103	30	26	27	59	40	12	173
Virion ELISA binding supernatants	313	250	204	137	109	215	185	56	318
Cloned antibodies									
Neutralizing antibodies	29	59	13	26	24	70	n/c [2]	n/c	n/c
Binding antibodies	25	19	18	10	18	21	n/c	n/c	n/c

[1] n/a, not applicable, not counted due to cell contamination;
[2] n/c, not cloned.

Supplementary Table 3. Functional and genetic properties of V160 induced antibodies

			Neutralization-IC50 (μg/ml)		Binding-EC50 (μg/ml)	Binding-OD450								Germline	
mAb	Subject	Target	ARPE-19	MRC-5	HCMV Virion	Trimer	Pentamer	gHgL	gB	pp65	pp28	pp71	pp150	Heavy	Light
1	300105	gHgL	0.1343	0.2951	0.1116	1.9258	2.0241	1.2879	N.B. [2]	N.B.	N.B.	N.B.	N.B.	HV1-18	KV1-5
2	300105	gHgL	0.0602	0.5138	0.0713	0.9854	1.5548	1.1689	N.B.	N.B.	N.B.	N.B.	N.B.	HV1-18	KV1-12
3	300105	gHgL	0.0846	0.5641	0.0838	1.4587	1.2548	1.0868	N.B.	N.B.	N.B.	N.B.	N.B.	HV3-9	KV1-5
4	300105	gHgL	0.0405	0.263	0.0949	1.6987	1.7471	1.7539	N.B.	N.B.	N.B.	N.B.	N.B.	HV1-18	KV1-5
5	300105	gHgL	0.0232	0.1021	0.1312	0.9863	1.115	1.3614	N.B.	N.B.	N.B.	N.B.	N.B.	HV3-11	KV3-11
6	300105	Pentamer-specific	0.0771	N.N. [1]	0.3613	N.B.	1.2548	N.B.	N.B.	N.B.	N.B.	N.B.	N.B.	HV1-18	KV2D-29
7	300105	gHgL	0.0366	N.N.	0.0654	1.5698	1.5078	1.5117	N.B.	N.B.	N.B.	N.B.	N.B.	HV1-18	KV1-5
8	300105	gHgL	0.1168	0.279	0.1089	0.9865	1.3676	1.0416	N.B.	N.B.	N.B.	N.B.	N.B.	HV1-18	KV1-5
9	300105	gHgL	0.0385	1.3676	0.0893	1.3654	1.4019	1.8351	N.B.	N.B.	N.B.	N.B.	N.B.	HV1-18	KV1-27
10	300105	gHgL	0.037	0.2972	0.0853	1.8449	2.0154	1.8198	N.B.	N.B.	N.B.	N.B.	N.B.	HV3-7	KV1-39
11	300105	gHgL	0.0577	1.3	0.0908	1.0258	1.8521	1.3719	N.B.	N.B.	N.B.	N.B.	N.B.	HV1-18	KV1-5
12	300105	gHgL	0.1293	N.N.	0.4576	1.3584	1.1772	1.4739	N.B.	N.B.	N.B.	N.B.	N.B.	HV3-30	LV2-23
13	300105	gHgL	0.0338	0.3932	0.1317	1.6854	1.5765	1.594	N.B.	N.B.	N.B.	N.B.	N.B.	HV4-59	KV3-20
14	300105	gHgL	0.1417	0.9264	0.1632	1.5632	1.8078	1.7853	N.B.	N.B.	N.B.	N.B.	N.B.	HV1-18	KV1-5
15	300105	gHgL	0.067	0.405	0.0802	1.3564	1.9242	1.8944	N.B.	N.B.	N.B.	N.B.	N.B.	HV1-18	KV1-12
16	300105	gHgL	0.0549	0.3737	0.0796	1.5632	1.2874	1.6324	N.B.	N.B.	N.B.	N.B.	N.B.	HV1-18	KV1-5
17	300105	gHgL	0.2312	1.1742	0.1822	1.1254	1.3794	1.4573	N.B.	N.B.	N.B.	N.B.	N.B.	HV1-18	KV1-5
19	300105	gHgL	0.0683	0.2417	0.0999	1.9658	1.8818	1.7859	N.B.	N.B.	N.B.	N.B.	N.B.	HV1-18	KV1-5
20	300105	gHgL	6.6667	N.N.	1.967	0.8932	1.1012	1.0205	N.B.	N.B.	N.B.	N.B.	N.B.	HV3-30	LV3-21
21	300105	gHgL	0.0292	N.N.	0.2702	1.2365	1.7394	1.6563	N.B.	N.B.	N.B.	N.B.	N.B.	HV3-33	KV1-39
22	300105	gHgL	0.0723	N.N.	0.0711	2.2027	2.5555	2.0856	N.B.	N.B.	N.B.	N.B.	N.B.	HV1-18	KV1-5
23	300105	gHgL	0.3849	N.N.	0.2007	0.8622	0.5439	0.9338	N.B.	N.B.	N.B.	N.B.	N.B.	HV3-15	KV2-24
24	300105	gHgL	0.0498	0.1467	0.0554	1.2358	1.4857	1.4459	N.B.	N.B.	N.B.	N.B.	N.B.	HV1-18	KV1-5
25	300105	gHgL	0.0606	1.0228	0.1748	2.7811	2.7311	2.1376	N.B.	N.B.	N.B.	N.B.	N.B.	HV3-48	KV3-20
26	300089	gHgL	0.2185	0.3604	0.3836	1.8542	1.697	2.0958	N.B.	N.B.	N.B.	N.B.	N.B.	HV7-4-1	LV2-14
27	300089	Pentamer-specific	0.0068	N.N.	0.0723	N.B.	1.4947	N.B.	N.B.	N.B.	N.B.	N.B.	N.B.	HV7-4-1	LV3-25
36	300089	gHgL	0.3236	0.6426	0.2914	1.4251	1.3224	1.5837	N.B.	N.B.	N.B.	N.B.	N.B.	HV1-18	LV7-46
37	300089	gHgL	0.1297	0.3287	0.2891	1.6986	1.9178	1.9501	N.B.	N.B.	N.B.	N.B.	N.B.	HV4-31	KV3-20
41	300089	gHgL	1.8631	2.778	0.8502	1.5627	1.5839	1.6428	N.B.	N.B.	N.B.	N.B.	N.B.	HV1-2	LV2-14
42	300089	gHgL	0.139	N.N.	0.3463	1.1247	0.8462	0.9408	N.B.	N.B.	N.B.	N.B.	N.B.	HV3-33	KV1-39
43	300089	gHgL	0.1626	0.5009	0.3448	1.3652	1.5967	1.5666	N.B.	N.B.	N.B.	N.B.	N.B.	HV4-31	KV3-20
56	300089	gHgL	0.0337	N.N.	0.0675	1.3654	1.2501	1.4774	N.B.	N.B.	N.B.	N.B.	N.B.	HV1-46	KV4-1
59	300105	gHgL	N.N.	N.N.	4.86	1.0666	0.9681	0.893	N.B.	N.B.	N.B.	N.B.	N.B.	HV3-7	LV2-14
62	300105	gHgL	N.N.	N.N.	0.0733	0.4986	0.656	0.3248	N.B.	N.B.	N.B.	N.B.	N.B.	HV3-21	KV3-20
65	300105	gB	N.N.	N.N.	0.0033	N.B.	N.B.	N.B.	2.3156	N.B.	N.B.	N.B.	N.B.	HV4-59	KV4-1
69	300105	gHgL	N.N.	N.N.	0.3627	0.5976	0.446	0.2954	N.B.	N.B.	N.B.	N.B.	N.B.	HV3-48	LV1-44

71	300105	gHgL	N.N.	N.N.	53.74	1.1164	1.1398	1.1591	N.B.	N.B.	N.B.	N.B.	N.B.	HV1-18	KV1-5
80	300105	gHgL	N.N.	N.N.	0.2919	0.971	0.9936	1.532	N.B.	N.B.	N.B.	N.B.	N.B.	HV5-51	LV3-1
83	300105	gHgL	N.N.	N.N.	0.0986	0.9684	1.2667	1.0829	N.B.	N.B.	N.B.	N.B.	N.B.	HV3-30	LV2-14
84	300105	gHgL	N.N.	N.N.	0.0676	1.6623	1.6253	0.9039	N.B.	N.B.	N.B.	N.B.	N.B.	HV4-59	KV1-39
104	300089	gHgL	N.N.	N.N.	0.0344	0.0805	0.0663	0.0971	N.B.	N.B.	N.B.	N.B.	N.B.	HV3-74	LV6-57
119	300089	gB	N.N.	N.N.	0.577	N.B.	N.B.	N.B.	1.1959	N.B.	N.B.	N.B.	N.B.	HV7-4-1	LV3-10
122	300089	gHgL	N.N.	N.N.	0.5303	0.3038	0.2543	0.3997	N.B.	N.B.	N.B.	N.B.	N.B.	HV3-30	KV3-15
123	300089	gHgL	N.N.	N.N.	0.0143	0.1759	0.259	0.6568	N.B.	N.B.	N.B.	N.B.	N.B.	HV3-53	KV1-5
129	300089	gHgL	N.N.	N.N.	0.0227	0.0853	0.0797	0.1138	N.B.	N.B.	N.B.	N.B.	N.B.	HV3-7	KV2-30
132	300089	gHgL	N.N.	N.N.	0.0316	0.0885	0.0765	0.0797	N.B.	N.B.	N.B.	N.B.	N.B.	HV3-64	LV6-57
134	300089	pp65	N.N.	N.N.	0.0337	N.B.	N.B.	N.B.	N.B.	0.8112	N.B.	N.B.	N.B.	HV3-11	KV2-24
136	300089	pp65	N.N.	N.N.	0.0444	N.B.	N.B.	N.B.	N.B.	2.1853	N.B.	N.B.	N.B.	HV3-11	KV2-24
138	300089	pp65	N.N.	N.N.	0.0633	N.B.	N.B.	N.B.	N.B.	1.2116	N.B.	N.B.	N.B.	HV3-74	LV3-1
139	300089	gHgL	N.N.	N.N.	0.5196	0.0798	0.1693	0.2413	N.B.	N.B.	N.B.	N.B.	N.B.	HV2-5	KV1-5
140	300089	pp65	N.N.	N.N.	0.0229	N.B.	N.B.	N.B.	N.B.	1.2907	N.B.	N.B.	N.B.	HV3-48	LV1-40
143	300089	pp28	N.N.	N.N.	0.035	N.B.	N.B.	N.B.	N.B.	N.B.	1.5015	N.B.	N.B.	HV3-15	KV2-28
147	300089	gHgL	N.N.	1.453	0.4394	1.1125	1.196	1.3012	N.B.	N.B.	N.B.	N.B.	N.B.	HV7-4-1	KV1-5
150	300089	gHgL	N.N.	0.8143	0.3445	2.2725	2.8748	1.6702	N.B.	N.B.	N.B.	N.B.	N.B.	HV1-2	LV2-23
151	300105	gB	N.N.	N.N.	0.0985	N.B.	N.B.	N.B.	1.1442	N.B.	N.B.	N.B.	N.B.	HV1-69	LV2-23
153	300105	gHgL	N.N.	N.N.	0.6194	1.1827	1.5795	0.9176	N.B.	N.B.	N.B.	N.B.	N.B.	HV4-39	LV2-14
160	300105	gHgL	N.N.	N.N.	0.7793	0.9775	0.9344	0.9562	N.B.	N.B.	N.B.	N.B.	N.B.	HV1-8	KV4-1
161	300105	pp65	N.N.	N.N.	0.0638	N.B.	N.B.	N.B.	N.B.	1.3026	N.B.	N.B.	N.B.	HV3-23	LV2-8
162	300105	gHgL	N.N.	N.N.	0.0209	0.9712	1.0715	1.1102	N.B.	N.B.	N.B.	N.B.	N.B.	HV3-13	LV2-14
165	300105	gB	N.N.	N.N.	0.4534	N.B.	N.B.	N.B.	2.362	N.B.	N.B.	N.B.	N.B.	HV1-69	KV4-1
166	300105	gHgL	N.N.	N.N.	0.0226	0.89	1.0972	1.3203	N.B.	N.B.	N.B.	N.B.	N.B.	HV3-21	KV3-15
169	300105	pp65	N.N.	N.N.	0.0002	N.B.	N.B.	N.B.	N.B.	1.2339	N.B.	N.B.	N.B.	HV1-18	LV2-11
170	300105	pp65	N.N.	N.N.	0.0306	N.B.	N.B.	N.B.	N.B.	1.4267	N.B.	N.B.	N.B.	HV4-59	LV1-51
171	300105	gB	N.N.	N.N.	0.1162	N.B.	N.B.	N.B.	1.1549	N.B.	N.B.	N.B.	N.B.	HV1-2	LV3-21
172	300105	gB	N.N.	N.N.	0.3076	N.B.	N.B.	N.B.	1.1607	N.B.	N.B.	N.B.	N.B.	HV5-51	LV2-14
173	300105	pp65	N.N.	N.N.	0.0104	N.B.	N.B.	N.B.	N.B.	1.1611	N.B.	N.B.	N.B.	HV4-4	KV1-5
175	300105	gB	N.N.	N.N.	0.0507	N.B.	N.B.	N.B.	1.15	N.B.	N.B.	N.B.	N.B.	HV3-74	LV9-49
201	300105	gHgL	34.459	N.N.	4.606	0.3734	0.8786	0.2864	N.B.	N.B.	N.B.	N.B.	N.B.	HV3-30	KV1-33
238	300103	gHgL	0.0543	0.1364	0.118	1.6548	1.5513	1.5574	N.B.	N.B.	N.B.	N.B.	N.B.	HV3-49	LV3-25
239	300103	gHgL	0.196	0.8194	0.6455	1.4665	0.7178	1.1616	N.B.	N.B.	N.B.	N.B.	N.B.	HV3-23	KV3-20
240	300103	gHgL	0.0258	N.N.	0.6356	0.8996	1.1687	1.2477	N.B.	N.B.	N.B.	N.B.	N.B.	HV3-21	KV3-15
242	300103	gHgL	0.0549	0.2974	1.293	1.1254	0.7619	0.9105	N.B.	N.B.	N.B.	N.B.	N.B.	HV3-30	KV1-5
244	300103	gHgL	0.0255	N.N.	0.0337	1.6542	1.0066	1.4561	N.B.	N.B.	N.B.	N.B.	N.B.	HV1-18	KV1-5
247	300103	gHgL	0.725	1.0506	1.879	0.9865	1.4225	1.4384	N.B.	N.B.	N.B.	N.B.	N.B.	HV3-30-3	LV2-14
250	300103	Pentamer-specific	0.0012	N.N.	0.0339	N.B.	1.5218	N.B.	N.B.	N.B.	N.B.	N.B.	N.B.	HV3-30-3	KV1-39
251	300103	gHgL	0.152	0.7505	1.225	1.6532	1.8851	1.7305	N.B.	N.B.	N.B.	N.B.	N.B.	HV4-34	LV2-14
252	300103	gHgL	0.0305	N.N.	0.127	1.3279	1.5562	1.6565	N.B.	N.B.	N.B.	N.B.	N.B.	HV1-18	KV1-5

256	300103	gHgL	1.4	0.5109	0.8316	1.3654	0.993	1.3442	N.B.	N.B.	N.B.	N.B.	N.B.	HV1-18	KV3-15
257	300103	gHgL	0.0279	N.N.	0.0378	2.3485	0.9703	1.536	N.B.	N.B.	N.B.	N.B.	N.B.	HV1-18	KV1-5
258	300103	gHgL	0.0301	0.727	0.4311	1.32546	1.5498	1.5102	N.B.	N.B.	N.B.	N.B.	N.B.	HV4-59	KV3-20
267	300103	gHgL	0.0233	N.N.	0.0509	1.2035	1.4355	1.5422	N.B.	N.B.	N.B.	N.B.	N.B.	HV4-39	LV5-45
269	300103	gHgL	0.0477	N.N.	2.592	0.7805	0.9312	1.4128	N.B.	N.B.	N.B.	N.B.	N.B.	HV3-15	KV2-28
279	300103	gHgL	0.0378	N.N.	0.5679	1.27	0.8804	1.2987	N.B.	N.B.	N.B.	N.B.	N.B.	HV3-23	KV1-5
286	300103	gHgL	0.347	N.N.	1.455	1.9938	2.0042	1.4676	N.B.	N.B.	N.B.	N.B.	N.B.	HV3-73	KV2-24
288	300103	gHgL	0.0188	N.N.	0.1865	1.7566	2.0188	1.3369	N.B.	N.B.	N.B.	N.B.	N.B.	HV3-15	KV2-24
292	300103	gHgL	3.09	N.N.	3.878	2.7624	1.9533	3.0687	N.B.	N.B.	N.B.	N.B.	N.B.	HV3-21	LV2-23
294	300103	gHgL	12.1	0.07904	11.62	2.7392	2.4457	0.4979	N.B.	N.B.	N.B.	N.B.	N.B.	HV3-23	KV3-20
295	300103	gHgL	0.137	5.549	3.148	0.772	1.0566	1.0991	N.B.	N.B.	N.B.	N.B.	N.B.	HV3-15	KV2-28
296	300103	gHgL	0.0496	N.N.	4.447	2.9639	1.9332	2.7824	N.B.	N.B.	N.B.	N.B.	N.B.	HV1-18	KV2-28
297	300103	gHgL	0.268	0.7935	0.275	1.5387	0.6768	0.9482	N.B.	N.B.	N.B.	N.B.	N.B.	HV3-7	KV1-12
298	300103	Pentamer-specific	0.0047	N.N.	2.35	N.B.	1.7424	N.B.	N.B.	N.B.	N.B.	N.B.	N.B.	HV1-18	KV1-5
300	300103	gHgL	0.0707	0.4165	0.1218	1.3695	1.6546	1.677	N.B.	N.B.	N.B.	N.B.	N.B.	HV1-18	KV1-5
302	300103	gHgL	0.0097	1.122	0.3753	1.2654	1.657	1.4695	N.B.	N.B.	N.B.	N.B.	N.B.	HV4-39	LV3-9
303	300103	gHgL	0.0454	1.153	0.1228	1.1298	1.4069	1.4968	N.B.	N.B.	N.B.	N.B.	N.B.	HV1-18	KV1-5
305	300103	Pentamer-specific	0.0182	N.N.	27.439	N.B.	1.4016	N.B.	N.B.	N.B.	N.B.	N.B.	N.B.	HV1-18	KV1-13
312	300103	gHgL	0.0201	N.N.	0.0684	1.7075	0.7449	1.1014	N.B.	N.B.	N.B.	N.B.	N.B.	HV7-4-1	KV1-5
315	300103	Pentamer-specific	0.0021	N.N.	0.3345	N.B.	0.6938	N.B.	N.B.	N.B.	N.B.	N.B.	N.B.	HV3-30	LV3-1
323	300089	gHgL	N.N.	N.N.	0.0013	2.8057	2.612	2.2218	N.B.	N.B.	N.B.	N.B.	N.B.	HV3-64D	LV7-46
327	300103	gHgL	0.0187	0.4713	0.1308	1.9209	1.6469	1.5988	N.B.	N.B.	N.B.	N.B.	N.B.	HV3-15	KV2-24
328	300103	gHgL	0.0105	0.03507	0.0466	1.3657	1.7693	1.816	N.B.	N.B.	N.B.	N.B.	N.B.	HV1-18	KV1-5
329	300103	gHgL	0.0361	N.N.	1.09	1.8311	2.4092	1.1092	N.B.	N.B.	N.B.	N.B.	N.B.	HV3-21	KV1-5
332	300103	gHgL	0.0406	0.1685	0.2479	2.284	0.9611	1.5285	N.B.	N.B.	N.B.	N.B.	N.B.	HV3-49	KV1-12
334	300103	Pentamer-specific	0.0085	N.N.	10.655	N.B.	2.0672	N.B.	N.B.	N.B.	N.B.	N.B.	N.B.	HV3-30	LV2-8
345	300103	gHgL	0.745	0.2887	0.2567	1.1387	0.9503	1.3831	N.B.	N.B.	N.B.	N.B.	N.B.	HV5-10-1	KV3-15
349	300103	Pentamer-specific	0.0695	N.N.	0.3875	N.B.	0.7817	N.B.	N.B.	N.B.	N.B.	N.B.	N.B.	HV3-21	LV3-21
354	300103	Pentamer-specific	0.0017	N.N.	0.842	N.B.	0.8463	N.B.	N.B.	N.B.	N.B.	N.B.	N.B.	HV3-15	LV1-40
355	300103	gHgL	0.0954	0.007076	0.2332	1.1258	1.2512	1.5004	N.B.	N.B.	N.B.	N.B.	N.B.	HV4-39	LV5-45
359	300103	gHgL	0.0218	N.N.	0.0393	1.3695	1.6466	1.7011	N.B.	N.B.	N.B.	N.B.	N.B.	HV7-4-1	KV1-5
360	300103	gHgL	11.0605	N.N.	1.185	0.2662	1.3217	0.2294	N.B.	N.B.	N.B.	N.B.	N.B.	HV3-30-3	KV3-20
362	300103	gHgL	0.3431	N.N.	7.78	1.1267	1.1983	1.205	N.B.	N.B.	N.B.	N.B.	N.B.	HV3-23	LV2-14
364	300103	gHgL	0.1535	N.N.	0.6481	0.7619	0.4665	0.6269	N.B.	N.B.	N.B.	N.B.	N.B.	HV3-15	KV3-20
366	300103	gHgL	1.9743	9.8349	6.051	0.3033	0.3406	0.3026	N.B.	N.B.	N.B.	N.B.	N.B.	HV1-24	LV2-8
367	300103	gHgL	3.0935	N.N.	9.022	1.7088	0.8469	1.195	N.B.	N.B.	N.B.	N.B.	N.B.	HV3-7	KV1-5
371	300103	gHgL	12.4281	N.N.	40.86	0.1235	0.1316	0.1267	N.B.	N.B.	N.B.	N.B.	N.B.	HV1-18	KV3-15
378	300103	gHgL	2.2338	N.N.	10.602	0.7642	0.5723	0.7037	N.B.	N.B.	N.B.	N.B.	N.B.	HV1-69	KV3-11
384	300103	Pentamer-specific	0.0042	N.N.	0.3012	N.B.	1.3257	N.B.	N.B.	N.B.	N.B.	N.B.	N.B.	HV3-30	LV2-14
406	300103	gB	N.N.	N.N.	0.1677	N.B.	N.B.	N.B.	1.5176	N.B.	N.B.	N.B.	N.B.	HV1-3	KV1-5
408	300103	gB	N.N.	N.N.	0.3442	N.B.	N.B.	N.B.	2.081	N.B.	N.B.	N.B.	N.B.	HV3-23	KV2-28

409	300103	gB	N.N.	N.N.	0.2062	N.B.	N.B.	N.B.	1.6066	N.B.	N.B.	N.B.	N.B.	HV2-70	KV1-5
416	300103	gB	N.N.	N.N.	0.0036	N.B.	N.B.	N.B.	2.2911	N.B.	N.B.	N.B.	N.B.	HV3-48	KV1-39
417	300103	gB	N.N.	N.N.	0.0011	N.B.	N.B.	N.B.	2.0741	N.B.	N.B.	N.B.	N.B.	HV1-2	KV3D-20
420	300103	gHgL	N.N.	N.N.	0.0306	0.0881	0.1236	0.0949	N.B.	N.B.	N.B.	N.B.	N.B.	HV3-23	LV3-21
422	300103	gB	N.N.	N.N.	0.0094	N.B.	N.B.	N.B.	2.0985	N.B.	N.B.	N.B.	N.B.	HV3-23	LV1-40
432	300103	gB	N.N.	N.N.	0.2137	N.B.	N.B.	N.B.	0.7321	N.B.	N.B.	N.B.	N.B.	HV4-39	KV1-5
443	300103	gHgL	N.N.	N.N.	0.1411	0.0962	0.1876	0.334	N.B.	N.B.	N.B.	N.B.	N.B.	HV6-1	KV2-28
450	300103	gB	N.N.	N.N.	0.1451	N.B.	N.B.	N.B.	1.9976	N.B.	N.B.	N.B.	N.B.	HV4-34	KV3D-20
457	300103	gB	N.N.	N.N.	0.0094	N.B.	N.B.	N.B.	2.58	N.B.	N.B.	N.B.	N.B.	HV1-18	KV3-11
459	300103	gB	N.N.	N.N.	0.0721	N.B.	N.B.	N.B.	2.0956	N.B.	N.B.	N.B.	N.B.	HV3-21	KV2-28
460	300103	gB	N.N.	N.N.	0.1172	N.B.	N.B.	N.B.	1.4445	N.B.	N.B.	N.B.	N.B.	HV3-23	KV3D-20
461	300103	gB	N.N.	N.N.	0.1289	N.B.	N.B.	N.B.	1.7693	N.B.	N.B.	N.B.	N.B.	HV3-30	KV1-17
486	300103	gB	N.N.	N.N.	0.1284	N.B.	N.B.	N.B.	2.5733	N.B.	N.B.	N.B.	N.B.	HV3-15	KV3-20
488	300103	gB	N.N.	N.N.	0.0708	N.B.	N.B.	N.B.	2.5314	N.B.	N.B.	N.B.	N.B.	HV3-30	KV1-39
532	300226	gHgL	0.1088	N.N.	2.135	0.0752	0.1908	0.0858	N.B.	N.B.	N.B.	N.B.	N.B.	HV2-70	KV4-1
534	300209	Pentamer-specific	0.0003	N.N.	N.B.	N.B.	1.9209	N.B.	N.B.	N.B.	N.B.	N.B.	N.B.	HV4-61	KV1-39
541	300209	gHgL	0.0016	0.4276	0.0368	1.7782	1.6469	1.7024	N.B.	N.B.	N.B.	N.B.	N.B.	HV1-18	KV1-5
548	300209	gHgL	0.0094	0.0495	0.0507	1.4294	0.5815	1.108	N.B.	N.B.	N.B.	N.B.	N.B.	HV3-43D	KV4-1
550	300209	gHgL	0.0101	0.6236	0.2218	1.7478	1.2184	1.4047	N.B.	N.B.	N.B.	N.B.	N.B.	HV3-30	KV1-16
554	300209	gHgL	0.0524	0.2438	0.297	1.6012	1.7643	1.8855	N.B.	N.B.	N.B.	N.B.	N.B.	HV4-30-4	KV3-20
557	300209	gHgL	0.0277	0.3426	0.0869	1.711	1.9459	1.8725	N.B.	N.B.	N.B.	N.B.	N.B.	HV4-59	KV3-20
565	300209	gHgL	0.2035	N.N.	0.0424	1.3009	1.5636	1.7994	N.B.	N.B.	N.B.	N.B.	N.B.	HV4-39	KV3-20
567	300209	gHgL	0.0232	0.1905	0.037	1.3658	1.535	1.5058	N.B.	N.B.	N.B.	N.B.	N.B.	HV1-18	KV1-5
570	300209	gHgL	0.0576	1.2113	0.1995	0.5687	0.6752	0.9117	N.B.	N.B.	N.B.	N.B.	N.B.	HV4-59	KV3-20
571	300209	gHgL	0.044	0.6538	0.0485	2.4861	1.2264	1.5988	N.B.	N.B.	N.B.	N.B.	N.B.	HV1-3	KV1-5
575	300209	gHgL	0.2726	0.9637	0.3051	0.8986	0.5282	0.6539	N.B.	N.B.	N.B.	N.B.	N.B.	HV1-2	LV2-14
577	300209	gHgL	0.0136	0.0944	0.0306	1.4698	1.4877	1.4061	N.B.	N.B.	N.B.	N.B.	N.B.	HV1-18	KV1-5
578	300209	gHgL	10.6254	N.N.	0.8068	1.6766	0.782	1.0124	N.B.	N.B.	N.B.	N.B.	N.B.	HV3-11	KV1-39
580	300209	gB	N.N.	N.N.	2.457	N.B.	N.B.	N.B.	2.5905	N.B.	N.B.	N.B.	N.B.	HV4-30-4	KV1-39
582	300209	gHgL	0.4026	N.N.	0.9092	0.9987	0.8188	0.952	N.B.	N.B.	N.B.	N.B.	N.B.	HV3-11	KV3-11
583	300209	Pentamer-specific	0.0008	N.N.	0.0099	N.B.	0.5736	N.B.	N.B.	N.B.	N.B.	N.B.	N.B.	HV3-30	LV1-36
584	300209	pp65	N.N.	N.N.	22.29	N.B.	N.B.	N.B.	N.B.	1.9303	N.B.	N.B.	N.B.	HV6-1	KV3-20
591	300227	gHgL	16.7953	N.N.	7.313	2.5367	2.5709	0.2571	N.B.	N.B.	N.B.	N.B.	N.B.	HV3-11	KV3-20
594	300227	gHgL	0.034	0.4837	0.0537	1.8652	1.6458	1.7893	N.B.	N.B.	N.B.	N.B.	N.B.	HV1-69	LV1-51
596	300227	gHgL	0.0408	0.4529	0.0718	1.4036	1.6862	1.7124	N.B.	N.B.	N.B.	N.B.	N.B.	HV1-69	LV1-51
597	300226	gHgL	1.7884	0.8107	0.3101	1.6832	1.7433	1.6632	N.B.	N.B.	N.B.	N.B.	N.B.	HV5-51	KV3-20
598	300226	gHgL	0.1578	N.N.	0.1906	1.1028	1.1431	1.1593	N.B.	N.B.	N.B.	N.B.	N.B.	HV3-30	LV1-47
604	300226	gHgL	0.0604	N.N.	6.929	1.2545	0.9995	1.0919	N.B.	N.B.	N.B.	N.B.	N.B.	HV3-11	LV1-40
605	300226	Pentamer-specific	0.0007	N.N.	0.0413	N.B.	1.0366	N.B.	N.B.	N.B.	N.B.	N.B.	N.B.	HV3-74	KV2-30
609	300226	gHgL	0.0494	1.033	1.243	1.944	0.9071	1.1852	N.B.	N.B.	N.B.	N.B.	N.B.	HV3-33	KV4-1
612	300226	Pentamer-specific	0.0012	N.N.	1.015	N.B.	1.8718	N.B.	N.B.	N.B.	N.B.	N.B.	N.B.	HV4-38-2	LV1-44

613	300226	gHgL	0.0254	N.N.	0.0514	1.3285	1.3615	1.4375	N.B.	N.B.	N.B.	N.B.	N.B.	HV1-18	KV1-5
617	300226	gHgL	5.4669	N.N.	38.444	0.4968	0.5321	0.6921	N.B.	N.B.	N.B.	N.B.	N.B.	HV1-8	LV2-14
620	300226	gHgL	0.0522	0.9641	1.139	1.4273	0.8436	1.1698	N.B.	N.B.	N.B.	N.B.	N.B.	HV3-11	LV2-14
622	300226	gHgL	0.0224	1.968	0.1753	2.3886	0.9651	1.5685	N.B.	N.B.	N.B.	N.B.	N.B.	HV3-30	KV1-NL1
625	300226	Pentamer-specific	0.2915	N.N.	4.877	N.B.	2.5814	N.B.	N.B.	N.B.	N.B.	N.B.	N.B.	HV4-61	KV1-NL1
628	300226	gHgL	0.0683	0.1734	8.993	0.6631	0.5857	0.9472	N.B.	N.B.	N.B.	N.B.	N.B.	HV3-21	KV3-15
631	300226	gHgL	0.0456	0.06779	0.1016	1.7662	0.6478	0.8443	N.B.	N.B.	N.B.	N.B.	N.B.	HV3-9	LV1-44
632	300226	gHgL	0.1591	N.N.	0.1195	2.1605	0.9973	1.3209	N.B.	N.B.	N.B.	N.B.	N.B.	HV3-23	KV1-12
635	300226	gHgL	0.0467	0.05141	0.048	1.4206	1.3069	1.3269	N.B.	N.B.	N.B.	N.B.	N.B.	HV1-18	KV1-5
640	300226	gHgL	0.0884	2.5	0.1273	0.6838	0.5778	0.9262	N.B.	N.B.	N.B.	N.B.	N.B.	HV1-18	KV1-39
643	300226	Pentamer-specific	0.0052	N.N.	0.4422	N.B.	1.1309	N.B.	N.B.	N.B.	N.B.	N.B.	N.B.	HV4-59	KV3-20
645	300226	gHgL	3.8346	0.2221	1.557	0.924	2.101	0.3471	N.B.	N.B.	N.B.	N.B.	N.B.	HV3-23	KV1-39
647	300226	gHgL	0.0269	0.1684	0.0326	1.5217	1.7127	1.7762	N.B.	N.B.	N.B.	N.B.	N.B.	HV1-18	KV1-5
648	300226	gHgL	0.0126	0.275	0.1862	1.7279	1.6777	1.7808	N.B.	N.B.	N.B.	N.B.	N.B.	HV5-51	KV3-15
651	300226	gHgL	0.0195	0.058	0.1536	1.6967	1.5737	1.5755	N.B.	N.B.	N.B.	N.B.	N.B.	HV5-51	KV3-15
662	300226	gHgL	0.028	0.2449	0.0351	1.4879	1.2911	1.3548	N.B.	N.B.	N.B.	N.B.	N.B.	HV1-18	KV1-5
666	300226	gHgL	0.5608	1.5515	0.5586	1.8594	1.0698	1.3069	N.B.	N.B.	N.B.	N.B.	N.B.	HV3-72	LV1-44
668	300226	gHgL	0.1535	N.N.	0.1012	0.9601	0.8684	1.1664	N.B.	N.B.	N.B.	N.B.	N.B.	HV1-18	KV3-20
676	300226	gHgL	0.0662	0.1953	0.0295	1.5392	1.1176	1.412	N.B.	N.B.	N.B.	N.B.	N.B.	HV1-18	KV1-5
682	300226	gHgL	0.0789	N.N.	0.2666	2.3888	2.003	2.5107	N.B.	N.B.	N.B.	N.B.	N.B.	HV4-30-4	LV1-40
685	300226	Pentamer-specific	8.5524	13.8	8.615	N.B.	1.0519	N.B.	N.B.	N.B.	N.B.	N.B.	N.B.	HV4-59	LV1-47
690	300226	gHgL	3.2376	N.N.	7.838	0.2912	1.2851	0.1247	N.B.	N.B.	N.B.	N.B.	N.B.	HV4-38-2	KV1-5
695	300226	Pentamer-specific	0.0016	N.N.	N.B.	N.B.	0.4206	N.B.	N.B.	N.B.	N.B.	N.B.	N.B.	HV1-69	LV3-21
702	300226	Pentamer-specific	0.0031	N.N.	0.1083	N.B.	0.9411	N.B.	N.B.	N.B.	N.B.	N.B.	N.B.	HV4-61	LV3-19
703	300226	gHgL	6.5587	N.N.	5.967	2.353	2.1648	2.3613	N.B.	N.B.	N.B.	N.B.	N.B.	HV3-48	KV1-12
708	300226	gHgL	1.5075	N.N.	16.957	1.1493	1.1633	1.5828	N.B.	N.B.	N.B.	N.B.	N.B.	HV1-69	KV3-20
710	300226	gHgL	0.0332	0.2164	0.3064	2.2154	1.9654	2.2759	N.B.	N.B.	N.B.	N.B.	N.B.	HV3-11	KV1-5
712	300226	gHgL	0.1157	0.9273	0.199	2.5014	1.0927	1.4882	N.B.	N.B.	N.B.	N.B.	N.B.	HV3-33	KV1-39
714	300226	Pentamer-specific	0.0061	N.N.	0.0553	N.B.	0.4089	N.B.	N.B.	N.B.	N.B.	N.B.	N.B.	HV3-7	LV1-44
716	300226	gHgL	2.7148	N.N.	33.579	1.8406	2.2862	0.2721	N.B.	N.B.	N.B.	N.B.	N.B.	HV4-34	LV1-44
722	300226	gHgL	0.1452	0.0651	0.3781	1.3698	1.2584	1.2642	N.B.	N.B.	N.B.	N.B.	N.B.	HV4-59	LV2-8
730	300226	gHgL	0.0248	N.N.	2.175	1.2323	0.8763	1.2597	N.B.	N.B.	N.B.	N.B.	N.B.	HV5-51	KV3-15
735	300226	Pentamer-specific	0.0028	N.N.	0.0323	N.B.	0.8635	N.B.	N.B.	N.B.	N.B.	N.B.	N.B.	HV3-30	LV2-14
742	300227	gHgL	0.1003	1.612	0.1885	0.9805	0.74	1.1819	N.B.	N.B.	N.B.	N.B.	N.B.	HV1-18	KV3-11
743	300227	gHgL	0.1485	2.2078	0.2498	0.6731	0.5885	1.0184	N.B.	N.B.	N.B.	N.B.	N.B.	HV1-18	KV3-11
746	300227	Pentamer-specific	0.0178	N.N.	4.254	N.B.	1.5185	N.B.	N.B.	N.B.	N.B.	N.B.	N.B.	HV3-7	LV6-57
749	300227	gHgL	0.0635	1.403	0.0399	1.2361	1.0504	1.1512	N.B.	N.B.	N.B.	N.B.	N.B.	HV1-18	KV1-5
756	300226	gHgL	3.7371	N.N.	7.296	1.0284	2.6371	0.3584	N.B.	N.B.	N.B.	N.B.	N.B.	HV3-9	KV1-39
760	300226	gHgL	0.8709	N.N.	0.4481	1.6464	0.6471	0.8465	N.B.	N.B.	N.B.	N.B.	N.B.	HV2-70	KV1-39
761	300226	Pentamer-specific	0.0192	N.N.	0.0477	N.B.	0.4963	N.B.	N.B.	N.B.	N.B.	N.B.	N.B.	HV3-15	LV2-8

765	300226	Pentamer-specific	0.0007	N.N.	0.0275	N.B.	2.4454	N.B.	N.B.	N.B.	N.B.	N.B.	N.B.	HV3-30	LV3-21
767	300226	gHgL	0.8022	N.N.	1.63	0.1519	0.8209	0.5347	N.B.	N.B.	N.B.	N.B.	N.B.	HV3-30	LV1-47
770	300226	gHgL	0.5797	N.N.	0.1877	2.0651	1.0911	1.3777	N.B.	N.B.	N.B.	N.B.	N.B.	HV1-69	KV1-9
774	300226	gHgL	0.0342	0.03922	0.0204	0.9512	0.7187	1.3853	N.B.	N.B.	N.B.	N.B.	N.B.	HV1-18	KV1-5
780	300226	gHgL	1.3532	5.305	27.231	1.1703	3.0972	0.3316	N.B.	N.B.	N.B.	N.B.	N.B.	HV1-69	LV3-21
784	300226	gHgL	7.8613	N.N.	17.961	2.5152	2.3713	1.3106	N.B.	N.B.	N.B.	N.B.	N.B.	HV4-34	KV4-1
790	300227	gHgL	7.3002	N.N.	no-binding	1.4885	2.9248	0.6584	N.B.	N.B.	N.B.	N.B.	N.B.	HV1-18	KV2-28
792	300227	Pentamer-specific	0.0043	N.N.	0.2758	N.B.	2.4747	N.B.	N.B.	N.B.	N.B.	N.B.	N.B.	HV2-5	KV2-28
795	300227	gHgL	0.0588	0.005949	4.426	2.9849	2.8806	2.9454	N.B.	N.B.	N.B.	N.B.	N.B.	HV3-11	KV3-20
801	300227	gHgL	0.0138	0.2573	0.0499	1.4362	1.3075	1.3485	N.B.	N.B.	N.B.	N.B.	N.B.	HV7-4-1	KV1-5
806	300227	gHgL	11.6764	N.N.	30.204	1.0449	2.479	0.3645	N.B.	N.B.	N.B.	N.B.	N.B.	HV4-59	KV3-20
807	300227	gHgL	0.0053	0.23	0.0764	1.4443	0.9256	1.2295	N.B.	N.B.	N.B.	N.B.	N.B.	HV3-30-3	KV3-11
813	300227	gHgL	0.5826	0.04788	1.709	2.0118	0.9837	1.248	N.B.	N.B.	N.B.	N.B.	N.B.	HV1-2	KV1-27
815	300227	gHgL	0.7369	N.N.	10.101	1.0325	1.8262	1.5473	N.B.	N.B.	N.B.	N.B.	N.B.	HV3-30-3	KV1-12
816	300227	gHgL	3.0649	N.N.	N.B.	0.9437	0.7206	0.9861	N.B.	N.B.	N.B.	N.B.	N.B.	HV1-18	KV3-11
822	300227	gHgL	0.1469	0.3532	0.7332	2.5373	2.182	2.3089	N.B.	N.B.	N.B.	N.B.	N.B.	HV5-51	LV4-60
824	300227	gHgL	0.0298	0.1974	9.275	0.6985	0.5519	1.1451	N.B.	N.B.	N.B.	N.B.	N.B.	HV3-30-3	KV3-11
826	300226	gHgL	0.1754	0.4	0.4424	0.6123	0.7321	0.9053	N.B.	N.B.	N.B.	N.B.	N.B.	HV4-34	KV3-20
832	300226	gHgL	0.0183	0.495	0.3546	1.2803	1.2314	1.8208	N.B.	N.B.	N.B.	N.B.	N.B.	HV4-31	KV3-11
839	300226	gHgL	12.2331	N.N.	14.72	0.4586	1.4962	0.2512	N.B.	N.B.	N.B.	N.B.	N.B.	HV4-59	KV3-20
843	300226	gHgL	0.0291	0.1334	0.0241	0.9843	0.9947	1.3037	N.B.	N.B.	N.B.	N.B.	N.B.	HV1-18	KV1-5
845	300209	gB	49.3	N.N.	0.7	N.B.	N.B.	N.B.	1.145	N.B.	N.B.	N.B.	N.B.	HV4-61	KV1-39
850	300209	pp65	N.N.	N.N.	0.092	N.B.	N.B.	N.B.	N.B.	1.9322	N.B.	N.B.	N.B.	HV3-21	KV1-5
851	300209	gHgL	N.N.	N.N.	0.0121	0.3592	0.331	0.3468	N.B.	N.B.	N.B.	N.B.	N.B.	HV3-30-3	LV3-21
855	300209	gHgL	0.0936	0.2281	0.1244	1.3132	1.1921	1.6379	N.B.	N.B.	N.B.	N.B.	N.B.	HV4-34	LV2-14
858	300209	gB	37	38	0.0075	N.B.	N.B.	N.B.	1.1496	N.B.	N.B.	N.B.	N.B.	HV4-30-4	KV3-20
862	300209	gB	11.8	14.7	0.043	N.B.	N.B.	N.B.	1.1493	N.B.	N.B.	N.B.	N.B.	HV5-51	LV2-14
864	300227	pp65	N.N.	N.N.	0.0272	N.B.	N.B.	N.B.	N.B.	1.2851	N.B.	N.B.	N.B.	HV3-74	LV2-14
865	300227	gHgL	N.N.	N.N.	0.1886	0.5012	0.5719	0.7204	N.B.	N.B.	N.B.	N.B.	N.B.	HV3-9	KV1-39
867	300227	pp65	N.N.	N.N.	0.0199	N.B.	N.B.	N.B.	N.B.	1.4502	N.B.	N.B.	N.B.	HV3-11	KV1-39
868	300227	gHgL	1.2077	1.2023	0.4316	2.2245	0.8613	1.3267	N.B.	N.B.	N.B.	N.B.	N.B.	HV3-30	KV1-5
873	300227	pp65	N.N.	N.N.	0.0047	N.B.	N.B.	N.B.	N.B.	0.9691	N.B.	N.B.	N.B.	HV1-69-2	LV3-19
874	300227	gB	N.N.	N.N.	0.0665	N.B.	N.B.	N.B.	1.0769	N.B.	N.B.	N.B.	N.B.	HV4-39	KV3-20
883	300227	gHgL	N.N.	N.N.	0.067	1.6863	0.9975	0.9519	N.B.	N.B.	N.B.	N.B.	N.B.	HV4-30-4	KV1-39
885	300227	pp65	N.N.	N.N.	0.0195	N.B.	N.B.	N.B.	N.B.	1.4498	N.B.	N.B.	N.B.	HV4-34	KV1-39
891	300227	pp65	N.N.	N.N.	0.3862	N.B.	N.B.	N.B.	N.B.	1.5731	N.B.	N.B.	N.B.	HV1-18	LV1-47
892	300227	gB	N.N.	N.N.	0.0007	N.B.	N.B.	N.B.	1.1129	N.B.	N.B.	N.B.	N.B.	HV4-38-2	KV3-11
896	300227	pp65	N.N.	N.N.	0.0019	N.B.	N.B.	N.B.	N.B.	1.5994	N.B.	N.B.	N.B.	HV3-23	LV1-40
897	300227	pp65	N.N.	N.N.	0.005	N.B.	N.B.	N.B.	N.B.	1.5214	N.B.	N.B.	N.B.	HV3-23	LV1-40
898	300227	pp65	N.N.	N.N.	0.0036	N.B.	N.B.	N.B.	N.B.	1.5441	N.B.	N.B.	N.B.	HV3-23	LV1-40

899	300227	pp65	N.N.	N.N.	0.0204	N.B.	N.B.	N.B.	N.B.	2.147	N.B.	N.B.	N.B.	HV4-39	KV3-20
904	300226	pp65	N.N.	N.N.	0.0154	N.B.	N.B.	N.B.	N.B.	1.1459	N.B.	N.B.	N.B.	HV4-34	LV1-44
905	300226	gHgL	N.N.	N.N.	0.4358	2.7971	3.1183	1.4017	N.B.	N.B.	N.B.	N.B.	N.B.	HV1-8	LV2-23
913	300226	gHgL	0.9055	N.N.	0.2534	1.8363	0.929	1.1876	N.B.	N.B.	N.B.	N.B.	N.B.	HV3-23	LV1-47
921	300226	gHgL	3.7212	N.N.	4.782	0.6821	0.5673	0.9507	N.B.	N.B.	N.B.	N.B.	N.B.	HV4-4	KV3-11
928	300226	gB	N.N.	N.N.	0.0089	N.B.	N.B.	N.B.	1.5421	N.B.	N.B.	N.B.	N.B.	HV1-69	LV3-21
932	300226	pp65	N.N.	N.N.	0.052	N.B.	N.B.	N.B.	N.B.	1.3605	N.B.	N.B.	N.B.	HV1-69	KV1-8
936	300209	gB	N.N.	N.N.	0.1184	N.B.	N.B.	N.B.	1.4326	N.B.	N.B.	N.B.	N.B.	HV4-59	LV3-9
937	300209	pp65	N.N.	N.N.	0.1792	N.B.	N.B.	N.B.	N.B.	1.919	N.B.	N.B.	N.B.	HV1-69	KV1-16
940	300209	gHgL	0.029	0.8679	0.0323	2.4104	1.1589	1.4672	N.B.	N.B.	N.B.	N.B.	N.B.	HV1-69	KV1-5
945	300209	pp65	N.N.	N.N.	0.5114	N.B.	N.B.	N.B.	N.B.	1.2065	N.B.	N.B.	N.B.	HV4-30-4	KV1-39
955	300209	gB	N.N.	N.N.	0.0055	N.B.	N.B.	N.B.	1.132	N.B.	N.B.	N.B.	N.B.	HV4-61	KV4-1
957	300209	gB	N.N.	N.N.	0.0582	N.B.	N.B.	N.B.	1.1432	N.B.	N.B.	N.B.	N.B.	HV4-30-4	KV3-20
958	300227	pp65	N.N.	N.N.	0.081	N.B.	N.B.	N.B.	N.B.	1.3832	N.B.	N.B.	N.B.	HV1-69	KV3-15
959	300227	gB	N.N.	N.N.	0.3717	N.B.	N.B.	N.B.	1.1218	N.B.	N.B.	N.B.	N.B.	HV1-69	KV3-20
960	300227	gHgL	0.8357	N.N.	1.617	1.0297	0.9264	1.0831	N.B.	N.B.	N.B.	N.B.	N.B.	HV1-69	KV3-20
964	300227	pp65	N.N.	N.N.	0.054	N.B.	N.B.	N.B.	N.B.	1.2191	N.B.	N.B.	N.B.	HV3-9	LV1-44
966	300227	pp65	N.N.	N.N.	0.2665	N.B.	N.B.	N.B.	N.B.	1.5664	N.B.	N.B.	N.B.	HV4-34	KV1-39
977	300226	pp65	N.N.	N.N.	0.3174	N.B.	N.B.	N.B.	N.B.	1.9187	N.B.	N.B.	N.B.	HV4-31	LV3-1
980	300226	gB	N.N.	N.N.	0.2362	N.B.	N.B.	N.B.	1.196	N.B.	N.B.	N.B.	N.B.	HV3-64	LV3-21
988	300226	gHgL	N.N.	N.N.	0.1753	0.8257	0.6185	0.6347	N.B.	N.B.	N.B.	N.B.	N.B.	HV3-30-3	LV1-40
989	300226	gHgL	2.7254	N.N.	1.358	1.1336	1.2059	1.2315	N.B.	N.B.	N.B.	N.B.	N.B.	HV4-59	LV1-44
991	300226	pp65	N.N.	N.N.	0.0146	N.B.	N.B.	N.B.	N.B.	1.3241	N.B.	N.B.	N.B.	HV3-15	LV2-14
1023	300226	pp65	N.N.	N.N.	0.0236	N.B.	N.B.	N.B.	N.B.	2.1092	N.B.	N.B.	N.B.	HV1-18	LV4-60
1026	300226	gB	N.N.	N.N.	0.025	N.B.	N.B.	N.B.	1.0998	N.B.	N.B.	N.B.	N.B.	HV3-23	KV1-9
1034	300226	gB	N.N.	N.N.	0.091	N.B.	N.B.	N.B.	1.2725	N.B.	N.B.	N.B.	N.B.	HV3-33	LV3-21
1040	300226	gHgL	0.8	1.3652	3.45	0.6542	0.5672	1.1148	N.B.	N.B.	N.B.	N.B.	N.B.	HV4-61	KV3-20
1058	300226	gB	N.N.	N.N.	0.001	N.B.	N.B.	N.B.	1.1812	N.B.	N.B.	N.B.	N.B.	HV6-1	KV4-1
1071	300226	pp65	N.N.	N.N.	0.0061	N.B.	N.B.	N.B.	N.B.	1.2254	N.B.	N.B.	N.B.	HV3-15	LV1-51
1100	300226	gB	N.N.	N.N.	0.2943	N.B.	N.B.	N.B.	1.8229	N.B.	N.B.	N.B.	N.B.	HV3-11	LV3-21
1101	300226	gHgL	0.4885	N.N.	0.0248	0.5256	0.3993	0.1846	N.B.	N.B.	N.B.	N.B.	N.B.	HV4-30-4	KV3-20
1106	300226	gHgL	16.4709	N.N.	2.781	0.4126	0.8419	0.2644	N.B.	N.B.	N.B.	N.B.	N.B.	HV3-7	LV2-14
1114	300226	gHgL	2.6734	N.N.	8.577	0.7015	2.5859	0.306	N.B.	N.B.	N.B.	N.B.	N.B.	HV4-39	LV1-44

[1] N.N. indicates no neutralizing activity.

[2] N.B. indicates no binding to the HCMV virion or HCMV antigens

Supplementary Table 4. Consolidation of binding site of pentamer-specific reference antibodies from two studies by pairwise competition ELISA[1]

Antibody 1[2]	Target	Pairwise inhibition (%) with reference antibody 2[3]			
		1-103	1-125	2-18	57.4
4N10	UL130/UL131A	7	15	-17	-20
10P3	UL130/UL131A	5	2	-22	2
8I21	gHgL/UL128/UL130	3	10	-22	26
7I13	UL128/ UL130/ UL131A	17	22	-5	5
2C12	UL128/ UL130/ UL131A	15	**85**	-11	15
15D8	UL128	2	5	-8	**69**
10F7	UL130/UL131A	7	-1	-10	20

[1]ELISA plates coated with recombinant soluble gH pentamer were incubated with 3 µg/ml antibody 1 or PBS control prior exposure to 3 µg/ml biotinylated antibody 2. If antibody 1 and 2 compete for binding, the binding of antibody 2 will be decreased by the pretreated antibody 1 in comparison to the PBS control. The percentage of inhibition for each antibody 2 was calculated by normalizing the signal decrease to the binding signal in PBS control;

[2]Pentamer-specific antibodies from reference [2];

[3]Pentamer-specific antibodies from reference [3].

Supplementary Table 5. Mapping of binding sits of V160 induced pentamer-specific antibodies[1]

	Pairwise inhibition (%) with reference antibody 2[3]								
Antibody 1[2]	15D8	10F7	4N10	10P3	2C12	7I13	8I21	2-18	1-103
349	**74**	-2	2	4	0	0	1	-1	5
583	**69**	-6	-25	20	9	-6	2	20	22
761	**60**	-9	1	0	-2	-5	-8	-9	3
27	**58**	3	5	11	-3	6	-1	-2	5
765	9	**55**	-23	-11	28	-10	-16	-4	13
612	5	**51**	-19	0	12	-14	-9	8	20
298	3	**52**	-19	-29	10	-14	-23	-11	8
354	-2	0	-1	-1	**84**	5	-3	-5	17
695	4	ND[4]	ND	ND	**89**	ND	ND	9	-1
315	31	7	11	7	4	9	12	4	**54**
334	24	1	-22	-20	4	-6	-25	-24	**55**
735	17	-7	3	2	1	15	-7	-1	**58**
605	-13	ND	ND	ND	7	ND	ND	-4	**81**
702	24	ND	ND	ND	1	ND	ND	-7	**70**
6	23	-2	8	8	-2	12	1	5	6
714	22	4	-2	0	1	8	-12	-6	12
384	20	-1	4	10	-5	6	0	12	22
746	11	-4	3	3	2	5	4	-1	9
250	10	5	-17	-21	18	-5	-21	-10	11
625	4	3	-29	-23	5	-15	-24	-17	8
305	1	-3	18	2	-6	1	-1	4	1
643	-1	1	2	1	-1	-2	-2	0	-2
685	-2	-1	-26	-20	3	-20	-24	-12	8
20	-3	-3	4	1	-1	1	-3	6	0

[1]ELISA plates coated with recombinant soluble gH pentamer were incubated with 3 µg/ml antibody 1 or PBS control prior exposure to 3 µg/ml biotinylated antibody 2. If antibody 1 and 2 compete for binding, the binding of antibody 2 will be decreased by the pretreated antibody 1 in comparison to the PBS control. The percentage of inhibition for each antibody 2 was calculated by normalizing the signal decrease to the binding signal in PBS control

[2]V160 induced pentamer-specific antibodies;

[3]Reference pentamer-specific antibodies of known binding site;

[4]ND indicates the inhibition was not determined.

Supplementary Table 6. Mapping of binding sites of V160 induced gHgL antibodies[a]

Antibody 1[b]	Reference antibody 2[c]	Pairwise inhibition (%)
3	3-16	64
9	3-16	85
11	3-16	66
12	3-16	77
14	3-16	84
17	3-16	79
20	3-16	60
25	3-16	72
244	3-16	85
300	3-16	79
541	3-16	91
567	3-16	91
570	3-16	82
571	3-16	91
577	3-16	87
622	3-16	86
4	3-16	80
635	3-16	82
640	3-16	84
647	3-16	78
8	3-16	87
662	3-16	82
676	3-16	73
712	3-16	89
749	3-16	71
774	3-16	94
801	3-16	56

826	3-16	56
940	3-16	78
5	13H11	76
37	13H11	71
267	13H11	68
594	13H11	86
596	13H11	85
742	13H11	94
743	13H11	93
832	13H11	86
921	13H11	62
41	13H11	69
41	MSL-109	60
26	MSL-109	52
666	MSL-109	77
989	MSL-109	51
855	3G16	76

[a]ELISA plates coated with recombinant soluble gH/gL were incubated with 3 µg/ml antibody 1 or PBS control prior exposure to 3 µg/ml biotinylated antibody 2. If antibody 1 and 2 compete for binding, the binding of antibody 2 will be decreased by the pretreated antibody 1 in comparison to the PBS control. The percentage of inhibition for each antibody 2 was calculated by normalizing the signal decrease to the binding signal in PBS control;

[b]V160 induced gHgL antibodies;

[c]Reference gHgL antibodies of known binding site [3, 4, 5].

Supplementary Dataset. The individual data points of the figures, the original uncutted gel pictures and the antibody sequences .

Reference

1. DeKosky BJ*, et al.* In-depth determination and analysis of the human paired heavy- and light-chain antibody repertoire. *Nature medicine* **21**, 86-91 (2015).

2. Ciferri C*, et al.* Antigenic Characterization of the HCMV gH/gL/gO and Pentamer Cell Entry Complexes Reveals Binding Sites for Potently Neutralizing Human Antibodies. *PLoS pathogens* **11**, e1005230 (2015).

3. Ha S*, et al.* Neutralization of Diverse Human Cytomegalovirus Strains Conferred by Antibodies Targeting Viral gH/gL/pUL128-131 Pentameric Complex. *Journal of virology* **91**, (2017).

4. Macagno A*, et al.* Isolation of human monoclonal antibodies that potently neutralize human cytomegalovirus infection by targeting different epitopes on the gH/gL/UL128-131A complex. *Journal of virology* **84**, 1005-1013 (2010).

5. Fouts AE*, et al.* Mechanism for neutralizing activity by the anti-CMV gH/gL monoclonal antibody MSL-109. *Proceedings of the National Academy of Sciences of the United States of America* **111**, 8209-8214 (2014).
